# Synthesis and spectral characterization of 2,2-diphenylethyl glucosinolate and HPLC-based reaction progress curve data for the enzymatic hydrolysis of glucosinolates by *Sinapis alba* myrosinase

**DOI:** 10.1016/j.dib.2016.11.086

**Published:** 2016-11-28

**Authors:** Chase A. Klingaman, Matthew J. Wagner, Justin R. Brown, John B. Klecker, Ethan H. Pauley, Colin J. Noldner, Jared R. Mays

**Affiliations:** Augustana University, Department of Chemistry, 2001 S. Summit Ave., Sioux Falls, SD 57197, USA

## Abstract

The data presented in this article are related to the research article, “HPLC-based enzyme kinetics assay for glucosinolate hydrolysis facilitate analysis of systems with both multiple reaction products and thermal enzyme denaturation” (C.K. Klingaman, M.J. Wagner, J.R. Brown, J.B. Klecker, E.H. Pauley, C.J. Noldner, J.R. Mays,) [1]. This data article describes (1) the synthesis and spectral characterization data of a non-natural glucosinolate analogue, 2,2-diphenylethyl glucosinolate, (2) HPLC standardization data for glucosinolate, isothiocyanate, nitrile, and amine analytes, (3) reaction progress curve data for enzymatic hydrolysis reactions with variable substrate concentration, enzyme concentration, buffer pH, and temperature, and (4) normalized initial velocities of hydrolysis/formation for analytes. These data provide a comprehensive description of the enzyme-catalyzed hydrolysis of 2,2-diphenylethyl glucosinolate (**5**) and glucotropaeolin (**6**) under widely varied conditions.

**Specifications Table**

**Table:** 

Subject area	Biochemistry
More specific subject area	Enzymology
Type of data	Synthetic experimentals/characterization, tables, graphs, figures
How data was acquired	NMR (JEOL ECS-400 400 MHz), IR (Nicolet Avatar FTIR), UV–vis (Shimadzu UV-2450 with TCC-240A cell chamber), HPLC (Agilent 1200 system with degasser, photodiode array detector, and temperature-controlled autosampler)
Data format	Analyzed
Experimental factors	All analytical standards and reagents were confirmed to be >95% purity
Experimental features	The synthesis and characterization of 2,2-diphenylethyl glucosinolate; HPLC standardization of glucosinolate, isothiocyanate, nitrile, and amine analytes; HPLC reaction progress curves for experiments with (1) variable substrate concentration, (2) variable enzyme concentration, (3) variable buffer pH, and (4) variable temperature; tables of initial velocities of hydrolysis/formation
Data source location	Sioux Falls, SD
Data accessibility	The data are available with this article.

**Value of the data**

• The experimental methods and characterization of 2,2-diphenylethyl glucosinolate and intermediates could be useful toward preparation of synthetic glucosinolates.
• HPLC standardization of glucosinolate, isothiocyanate, nitrile, and amine analytes could be useful toward individuals analyzing these compounds.
• Complete reaction progress curve datasets for enzymatic hydrolysis reactions conducted with variable experimental conditions provide a comprehensive dataset for this type of enzymatic transformation.
• Tables of normalized velocities of hydrolysis and product formation provide a complete, quantitative perspective of these enzymatic reactions.

## 1 Data

This article describes the synthesis and characterization data of the non-natural glucosinolate, 2,2-diphenylethyl glucosinolate (**5**), and data related to the kinetic analysis of this compound and glucotropaeolin (**6**) with *Sinapis alba* myrosinase. This body of data is related to the methodological innovations and enzymological studies described in the related article, “HPLC-based kinetics assay facilitates analysis of systems with multiple reactions components and thermal enzyme denaturation” [1]; to improve clarity, compound numbering from the related article has been retained.

The data presented in Fig. 1, Fig. 2, Fig. 3, Fig. 4, Fig. 5, Fig. 6, Fig. 7, Fig. 8, Fig. 9 describe the standardization of enzyme and analytes. Reaction progress curve data is provided for experiments evaluating the effects of variable substrate concentration (Fig. 10, Fig. 11, Fig. 12, Fig. 13, [Myr] = 8.83 U ml^−1^), variable enzyme concentration (Fig. 14), variable pH (Fig. 15, Fig. 16, Fig. 17, Fig. 18, [Myr] = 8.83 U ml^−1^; Fig. 19, Fig. 20, Fig. 21, [Myr] = 1.77 U ml^−1^), and variable temperature (Fig. 22, Fig. 23, Fig. 24, Fig. 25, [Myr] = 7.06 U ml^−1^; Fig. 26, Fig. 27, Fig. 28, [Myr] = 1.77 U ml^−1^). Reaction progress curve data was fit to the modified Lambert *W*(x) using nonlinear regression [1]. Initial rates were independently obtained from progress curves tracking analyte ([Gluc]_t_, [ITC]_t_, or [nitrile]_t_) at a specific wavelength, then normalized for the concentration of myrosinase (*V*_0_ [Myr]^−1^, min^−1^) [1]. Complete original datasets for each figure are provided as Supplementary material.

### 1.1 Determination of myrosinase specific activity

Fig. 1.

### 1.2 HPLC standardization of analytes

Fig. 2, Fig. 3, Fig. 4, Fig. 5, Fig. 6, Fig. 7, Fig. 8, Fig. 9.

### 1.3 Reaction progress curves

#### 1.3.1 Variable concentration of substrate

**Fig. 10:**
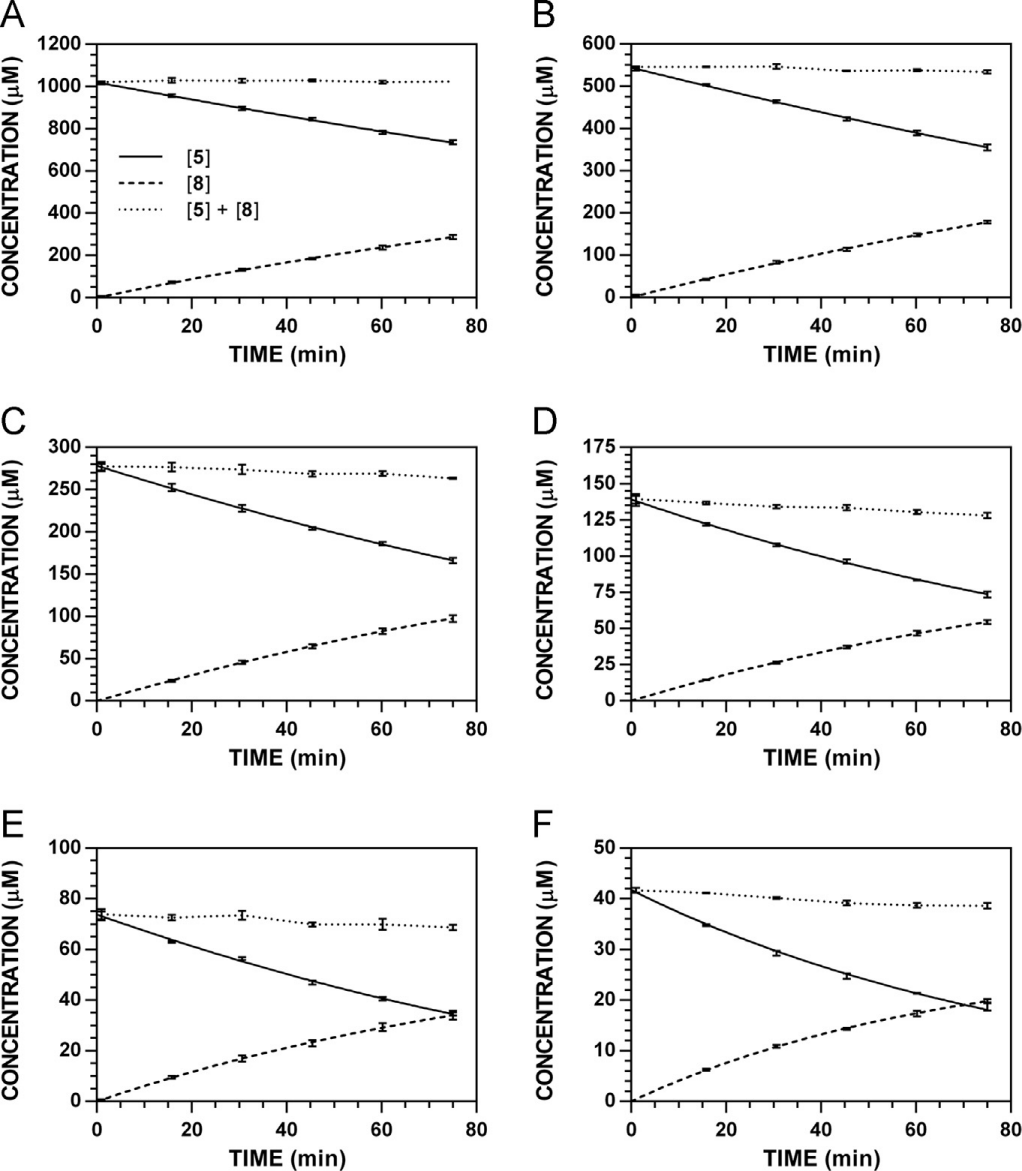
Reaction progress curves for the conversion of **5** to **8** at pH 7.4 and 37 °C (220 nm). **A.** [**5**]_0_ = 1000 μM. **B.** [**5**]_0_ = 500 μM. **C.** [**5**]_0_ = 250 μM. **D.** [**5**]_0_ = 125 μM. **E.** [**5**]_0_ = 62.5 μM. **F.** [**5**]_0_ = 31.3 μM.

**Fig. 11:**
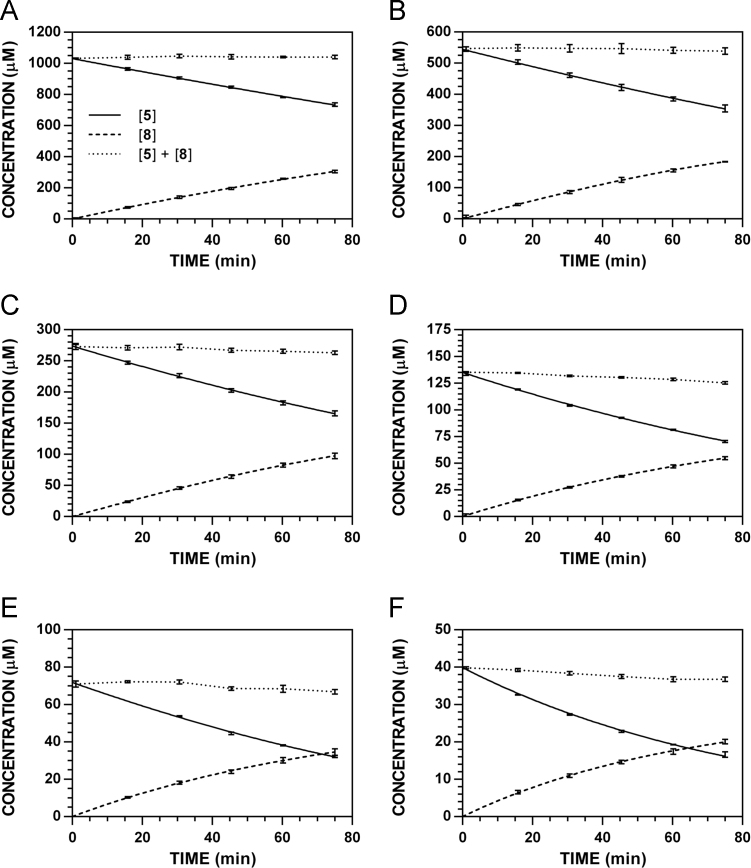
Reaction progress curves for the conversion of **5** to **8** at pH 7.4 and 37 °C (227 nm). **A.** [**5**]_0_ = 1000 μM. **B.** [**5**]_0_ = 500 μM. **C.** [**5**]_0_ = 250 μM. **D.** [**5**]_0_ = 125 μM. **E.** [**5**]_0_ = 62.5 μM. **F.** [**5**]_0_ = 31.3 μM. Panels A, C, and E appeared as representative data in C. A. Klingaman et. al and are included to provide a comprehensive perspective on this dataset (Fig. 2 in [1]).

**Fig. 12:**
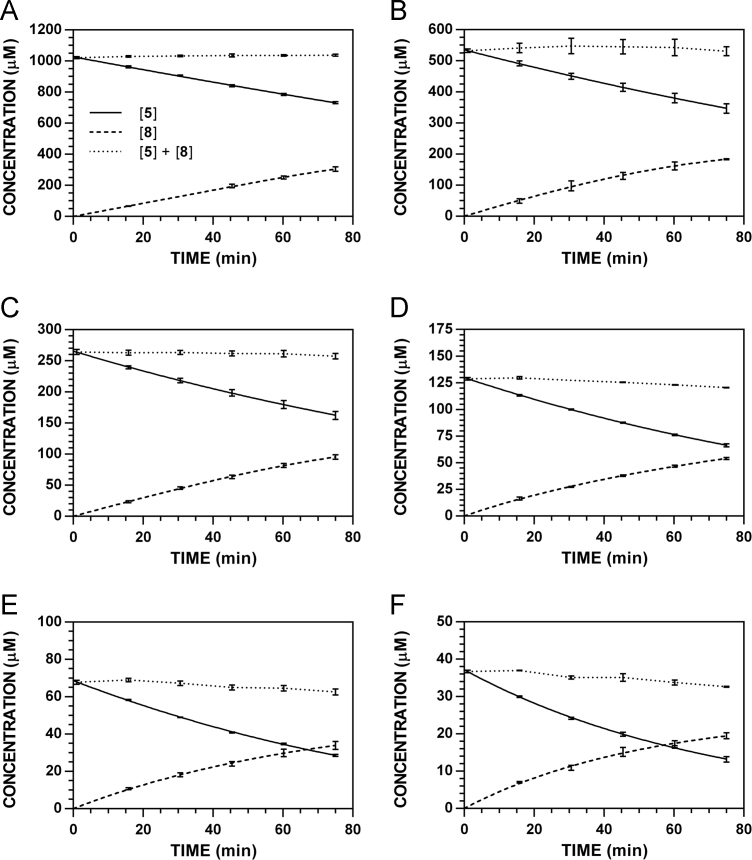
Reaction progress curves for the conversion of **5** to **8** at pH 7.4 and 37 °C (235 nm). **A.** [**5**]_0_ = 1000 μM. **B.** [**5**]_0_ = 500 μM. **C.** [**5**]_0_ = 250 μM. **D.** [**5**]_0_ = 125 μM. **E.** [**5**]_0_ = 62.5 μM. **F.** [**5**]_0_ = 31.3 μM.

**Fig. 13:**
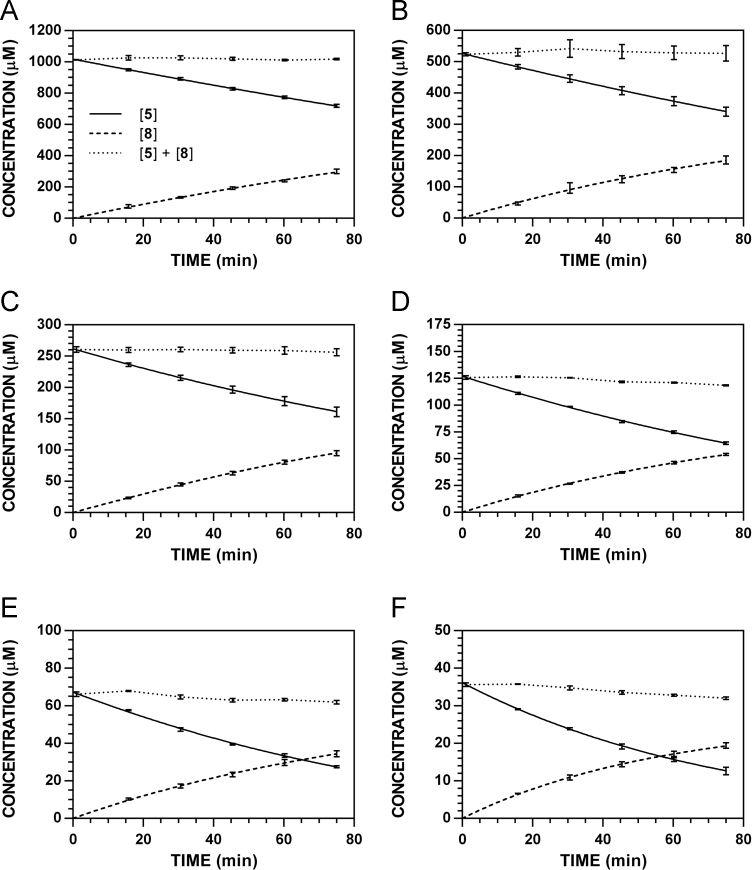
Reaction progress curves for the conversion of **5** to **8** at pH 7.4 and 37 °C (241 nm). **A.** [**5**]_0_ = 1000 μM. **B.** [**5**]_0_ = 500 μM. **C.** [**5**]_0_ = 250 μM. **D.** [**5**]_0_ = 125 μM. **E.** [**5**]_0_ = 62.5 μM. **F.** [**5**]_0_ = 31.3 μM.

Fig. 10, Fig. 11, Fig. 12, Fig. 13.

#### 1.3.2 Variable concentration of enzyme

Fig. 14.

#### 1.3.3 Variable pH

**Fig. 15:**
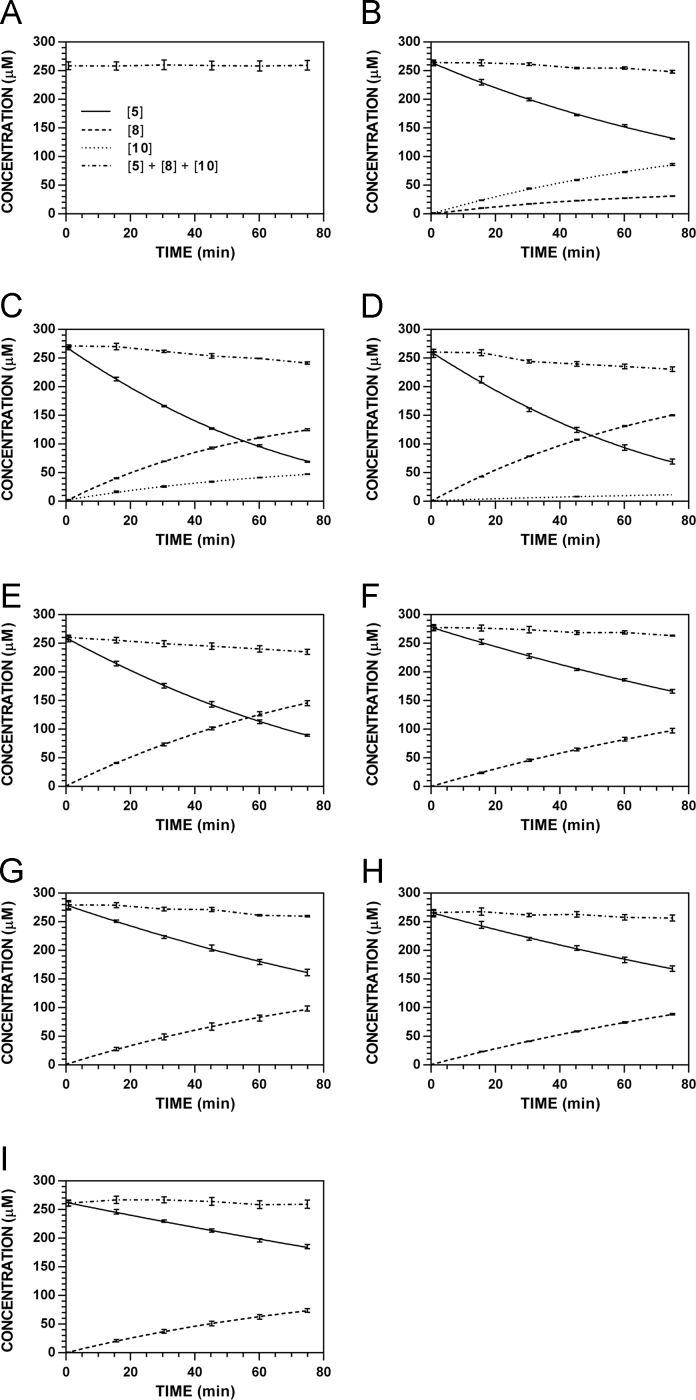
Reaction progress curves for the hydrolysis of **5** ([**5**]_0_ = 250 μM) at variable pH and 37 °C (220 nm). **A.** pH 2.0. **B.** pH 3.0. **C.** pH 4.0. **D.** pH 5.0. **E.** pH 6.0. **F.** pH 7.4. **G.** pH 8.0. **H.** pH 9.0. **I.** pH 10.0.

**Fig. 16:**
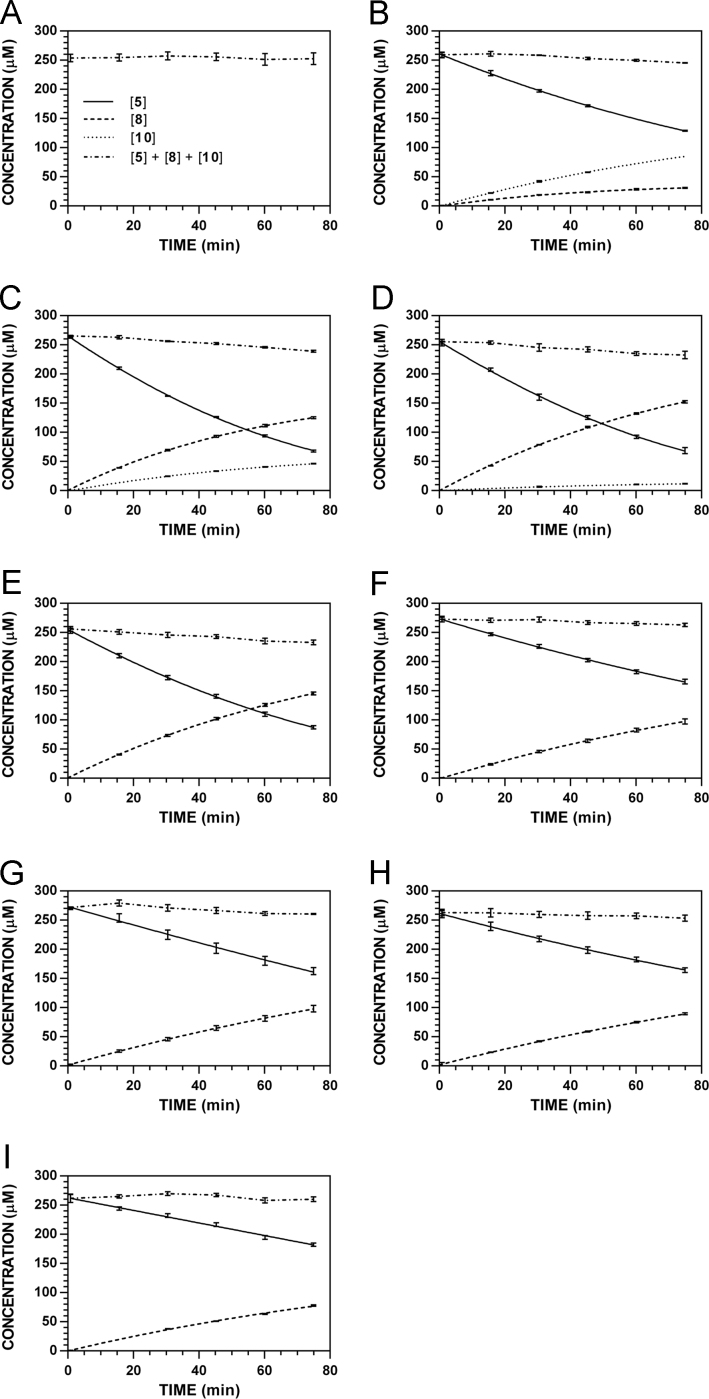
Reaction progress curves for the hydrolysis of **5** ([**5**]_0_ = 250 μM) at variable pH and 37 °C (227 nm). **A.** pH 2.0. **B.** pH 3.0. **C.** pH 4.0. **D.** pH 5.0. **E.** pH 6.0. **F.** pH 7.4. **G.** pH 8.0. **H.** pH 9.0. **I.** pH 10.0. Panels B, E, and I appeared as representative data in C. A. Klingaman et. al and are included to provide a comprehensive perspective on this dataset (Fig. 6 in [1]).

**Fig. 17:**
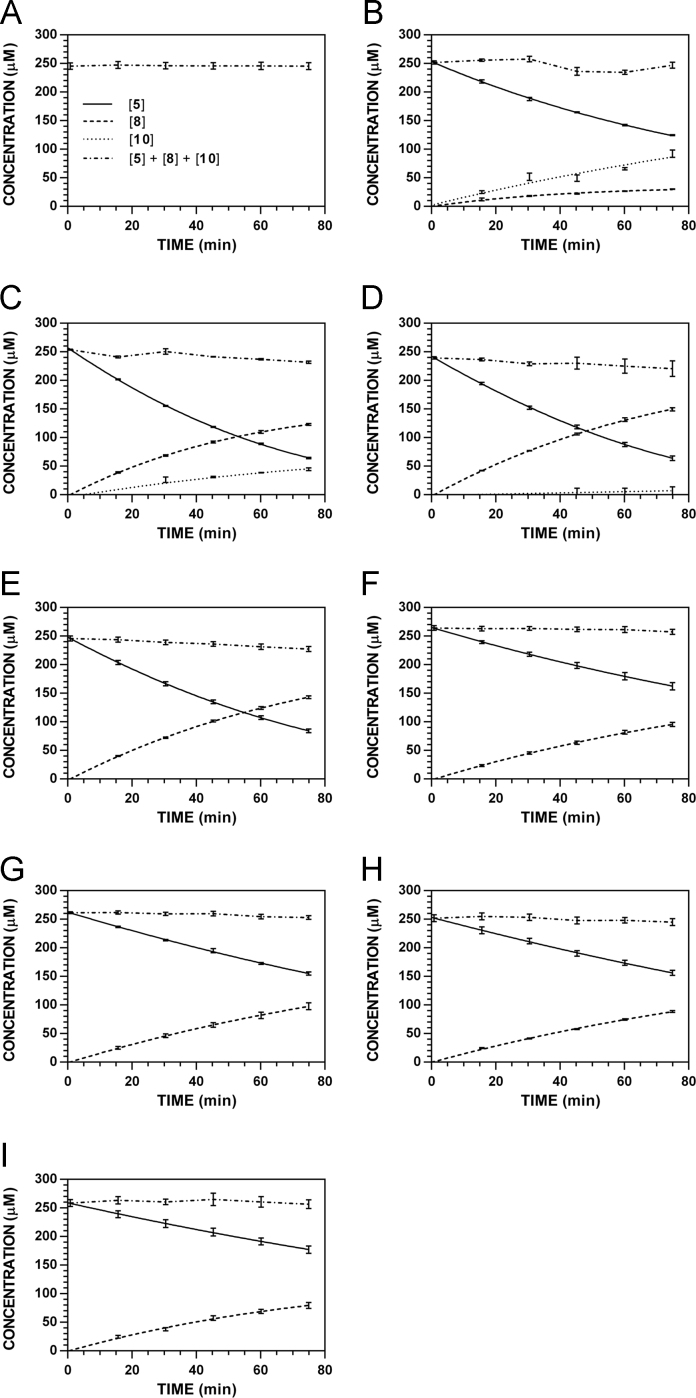
Reaction progress curves for the hydrolysis of **5** ([**5**]_0_ = 250 μM) at variable pH and 37 °C (235 nm). **A.** pH 2.0. **B.** pH 3.0. **C.** pH 4.0. **D.** pH 5.0. **E.** pH 6.0. **F.** pH 7.4. **G.** pH 8.0. **H.** pH 9.0. **I.** pH 10.0.

**Fig. 18:**
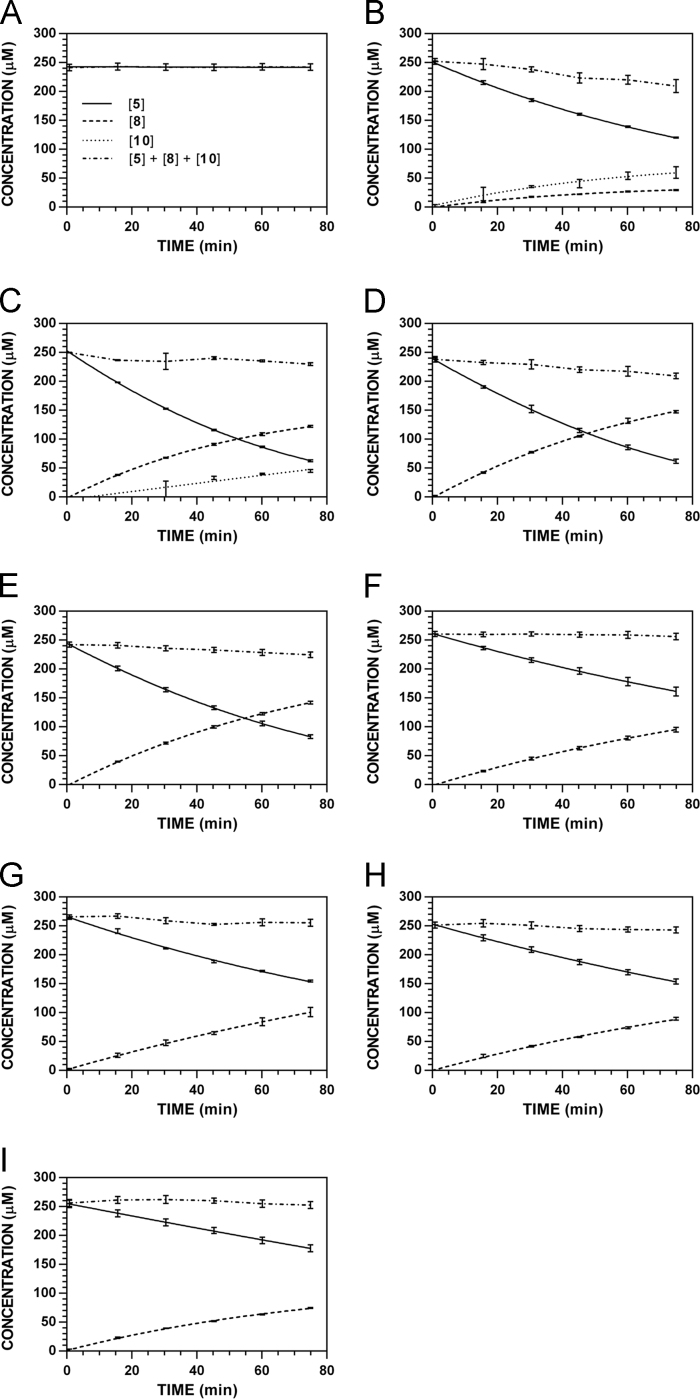
Reaction progress curves for the hydrolysis of **5** ([**5**]_0_ = 250 μM) at variable pH and 37 °C (241 nm). **A.** pH 2.0. **B.** pH 3.0. **C.** pH 4.0. **D.** pH 5.0. **E.** pH 6.0. **F.** pH 7.4. **G.** pH 8.0. **H.** pH 9.0. **I.** pH 10.0.

**Fig. 19:**
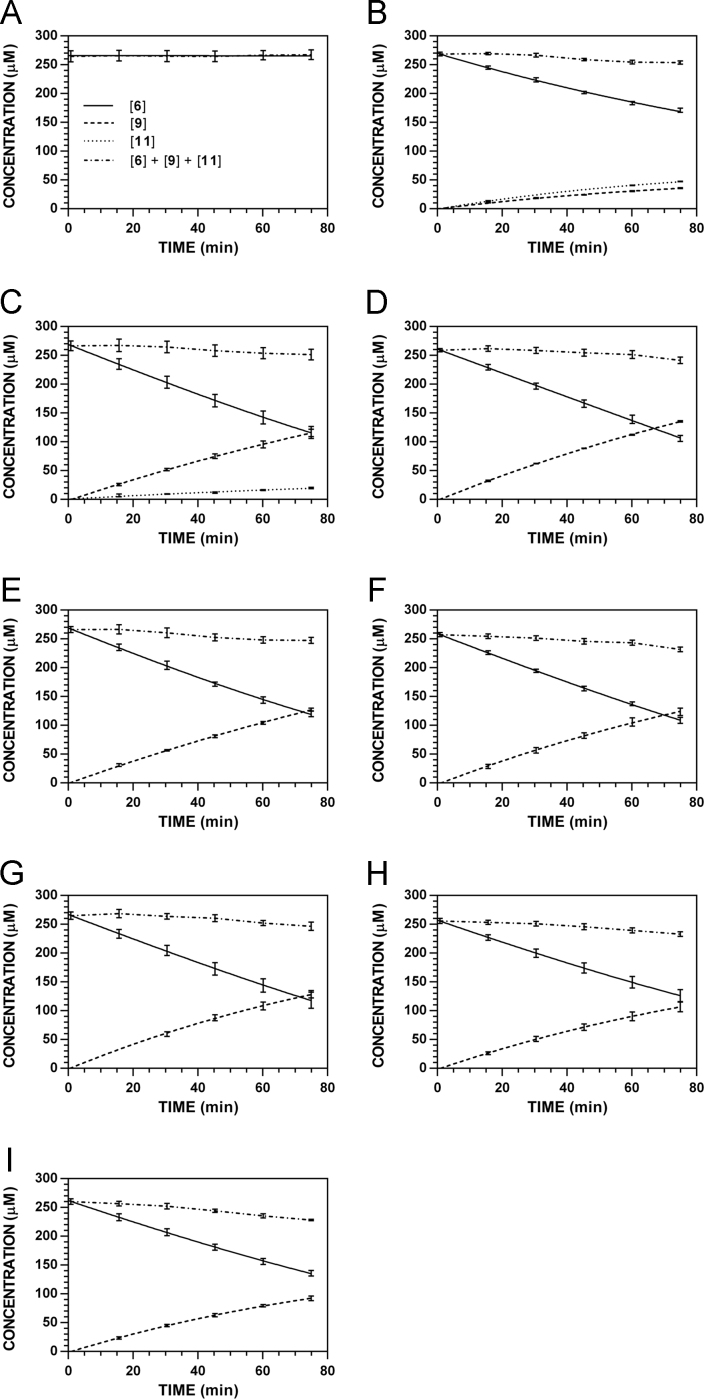
Reaction progress curves for the hydrolysis of **6** ([**6**]_0_ = 250 μM) at variable pH and 37 °C (**6**/**9**: 227 nm, **11**: 210 nm). **A.** pH 2.0. **B.** pH 3.0. **C.** pH 4.0. **D.** pH 5.0. **E.** pH 6.0. **F.** pH 7.4. **G.** pH 8.0. **H.** pH 9.0. **I.** pH 10.0. Panels B, E, and I appeared as representative data in C. A. Klingaman et. al and are included to provide a comprehensive perspective on this dataset (Fig. 6 in [1]).

**Fig. 20:**
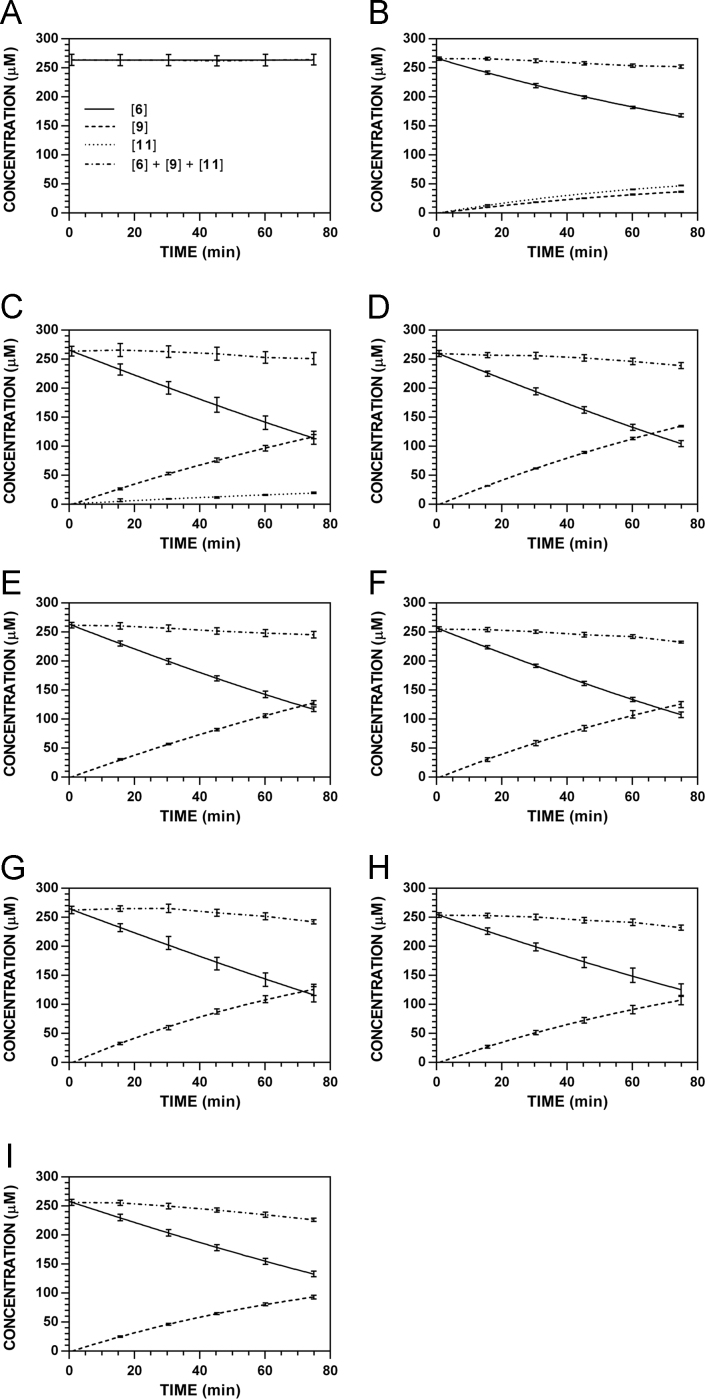
Reaction progress curves for the hydrolysis of **6** ([**6**]_0_ = 250 μM) at variable pH and 37 °C (**6**/**9**: 235 nm, **11**: 210 nm). **A.** pH 2.0. **B.** pH 3.0. **C.** pH 4.0. **D.** pH 5.0. **E.** pH 6.0. **F.** pH 7.4. **G.** pH 8.0. **H.** pH 9.0. **I.** pH 10.0.

**Fig. 21:**
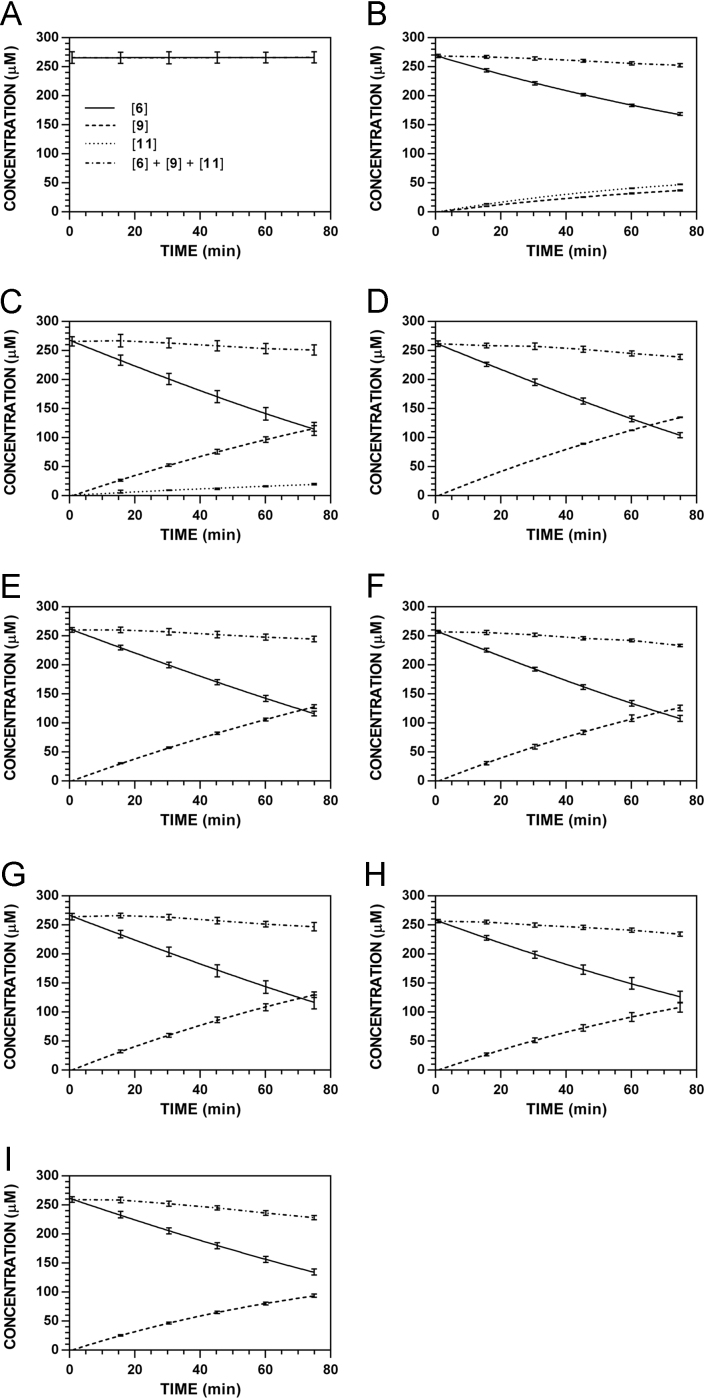
Reaction progress curves for the hydrolysis of **6** ([**6**]_0_ = 250 μM) at variable pH and 37 °C (**6**/**9**: 241 nm, **11**: 210 nm). **A.** pH 2.0. **B.** pH 3.0. **C.** pH 4.0. **D.** pH 5.0. **E.** pH 6.0. **F.** pH 7.4. **G.** pH 8.0. **H.** pH 9.0. **I.** pH 10.0.

Fig. 15, Fig. 16, Fig. 17, Fig. 18, Fig. 19, Fig. 20, Fig. 21.

#### 1.3.4 Variable temperature

**Fig. 22:**
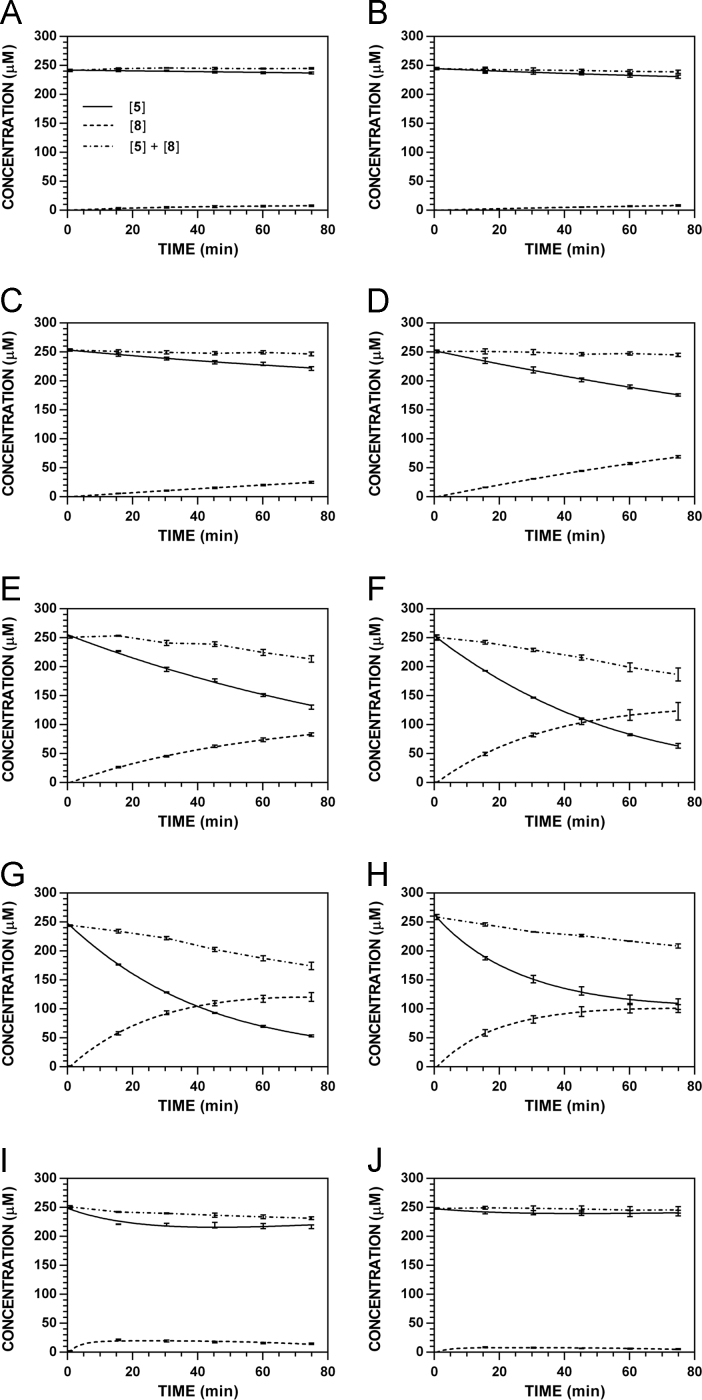
Reaction progress curves for the hydrolysis of **5** ([**5**]_0_ = 250 μM) at variable temperature and pH 7.4 (220 nm). **A.** 9 °C. **B.** 18 °C. **C.** 28.5 °C. **D.** 37 °C. **E.** 45 °C. **F.** 55 °C. **G.** 60 °C. **H.** 65 °C. **I.** 70 °C. **J.** 75 °C.

**Fig. 23:**
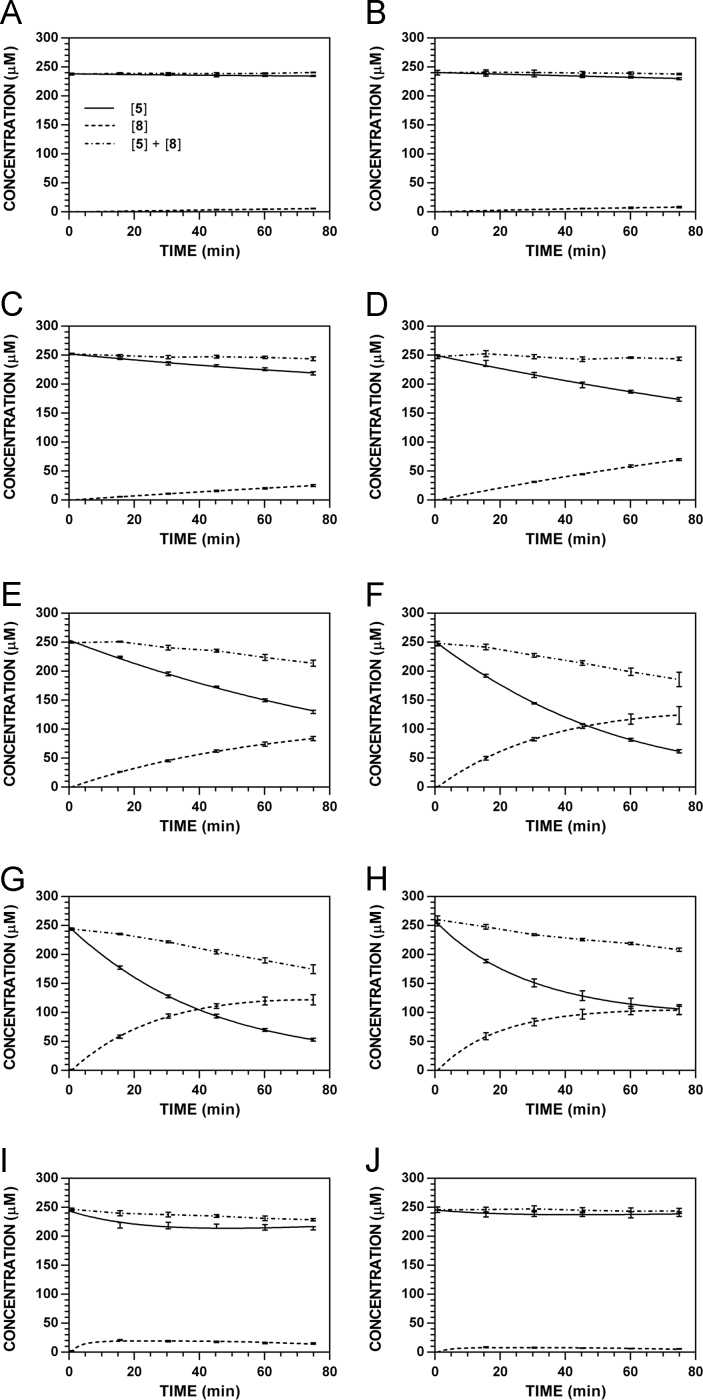
Reaction progress curves for the hydrolysis of **5** ([**5**]_0_ = 250 μM) at variable temperature and pH 7.4 (227 nm). **A.** 9 °C. **B.** 18 °C. **C.** 28.5 °C. **D.** 37 °C. **E.** 45 °C. **F.** 55 °C. **G.** 60 °C. **H.** 65 °C. **I.** 70 °C. **J.** 75 °C. Panels B, E, and H appeared as representative data in C. A. Klingaman et. al and are included to provide a comprehensive perspective on this dataset (Fig. 8 in [1]).

**Fig. 24:**
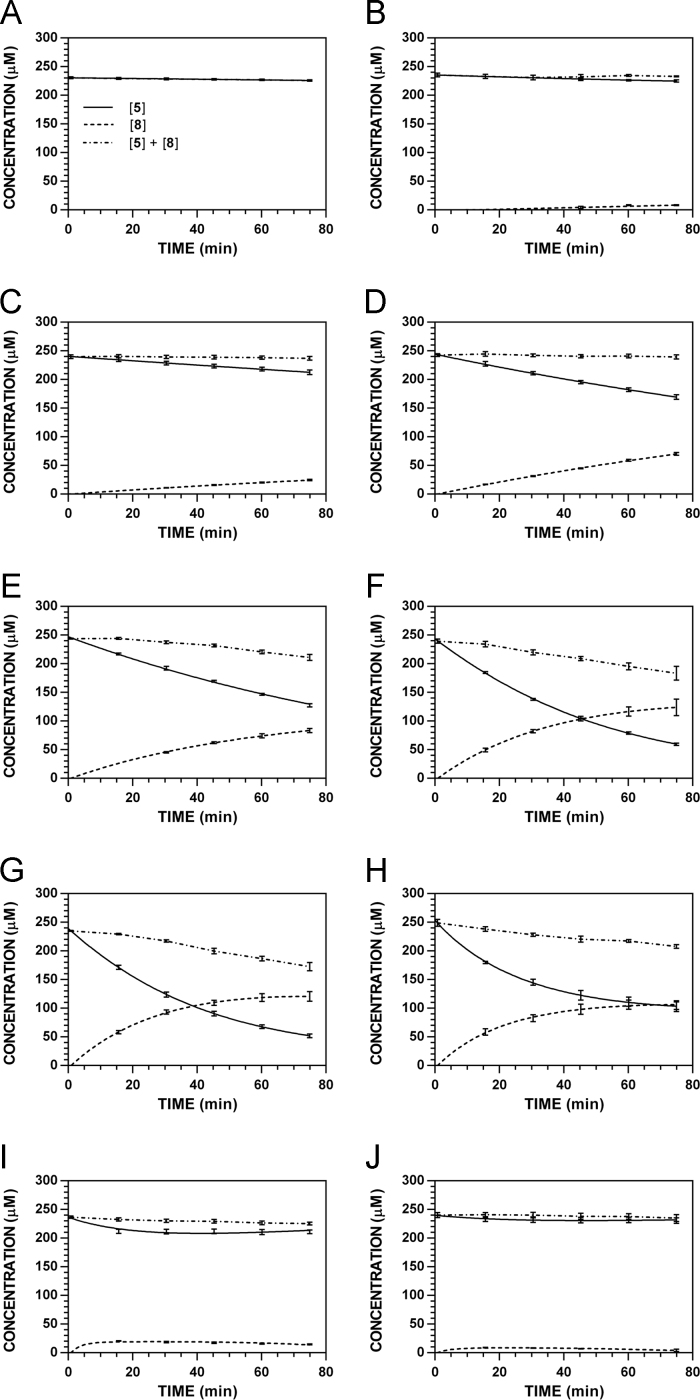
Reaction progress curves for the hydrolysis of **5** ([**5**]_0_ = 250 μM) at variable temperature and pH 7.4 (235 nm). **A.** 9 °C. **B.** 18 °C. **C.** 28.5 °C. **D.** 37 °C. **E.** 45 °C. **F.** 55 °C. **G.** 60 °C. **H.** 65 °C. **I.** 70 °C. **J.** 75 °C.

**Fig. 25:**
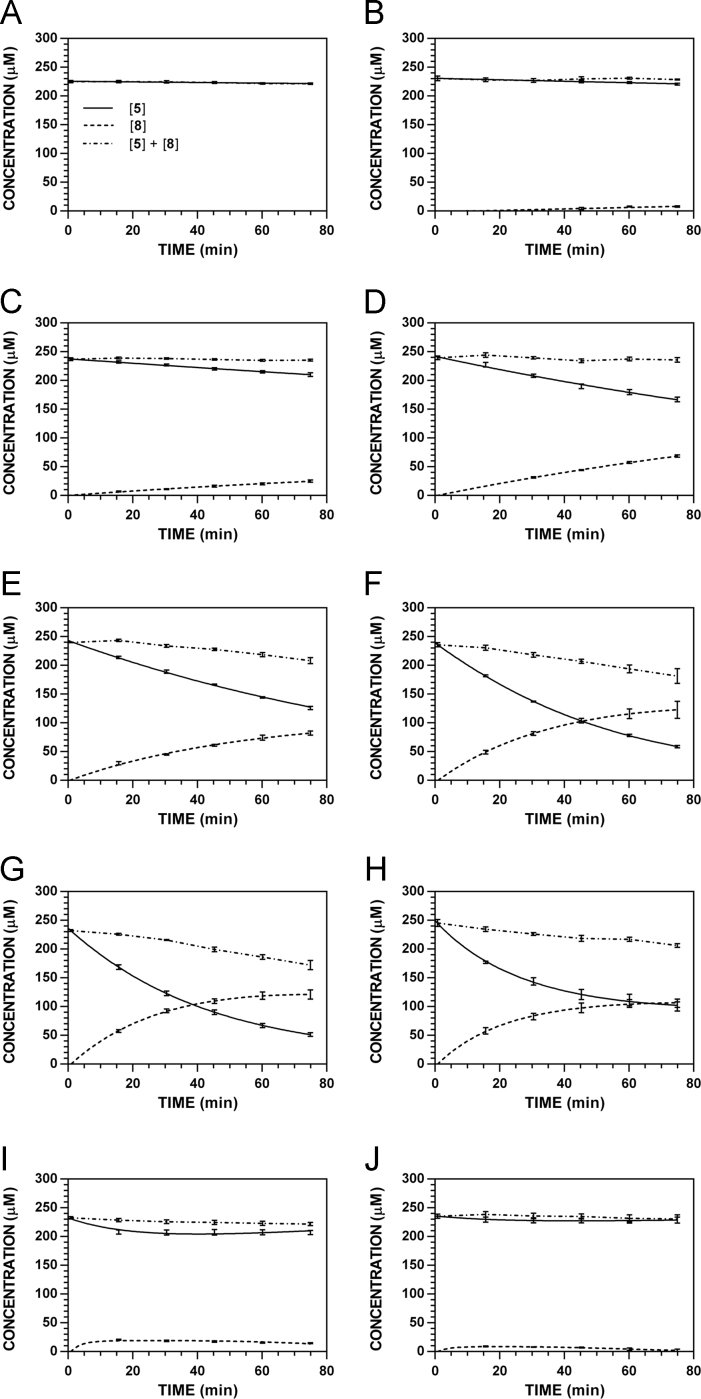
Reaction progress curves for the hydrolysis of **5** ([**5**]_0_ = 250 μM) at variable temperature and pH 7.4 (241 nm). **A.** 9 °C. **B.** 18 °C. **C.** 28.5 °C. **D.** 37 °C. **E.** 45 °C. **F.** 55 °C. **G.** 60 °C. **H.** 65 °C. **I.** 70 °C. **J.** 75 °C.

**Fig. 26:**
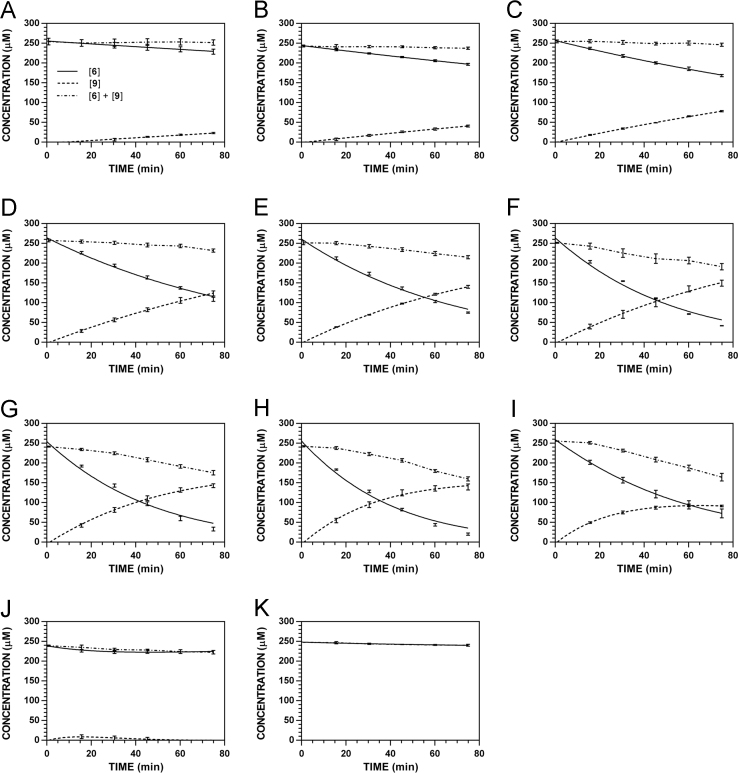
Reaction progress curves for the hydrolysis of **6** ([**6**]_0_ = 250 μM) at variable temperature and pH 7.4 (227 nm). **A.** 10.1 °C. **B.** 17.8 °C. **C.** 28.5 °C. **D.** 37 °C. **E.** 45 °C. **F.** 50 °C. **G.** 55 °C. **H.** 60 °C. **I.** 65 °C. **J.** 75 °C. **K.** 85 °C. Panels B, E, and I appeared as representative data in C. A. Klingaman et. al and are included to provide a comprehensive perspective on this dataset (Fig. 8 in [1]).

**Fig. 27:**
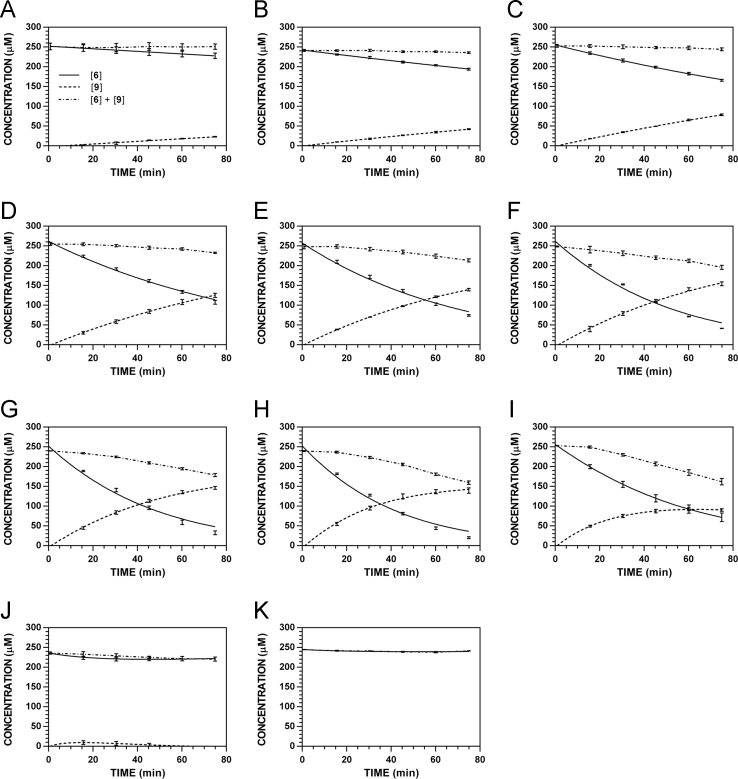
Reaction progress curves for the hydrolysis of **6** ([**6**]_0_ = 250 μM) at variable temperature and pH 7.4 (235 nm). **A.** 10.1 °C. **B.** 17.8 °C. **C.** 28.5 °C. **D.** 37 °C. **E.** 45 °C. **F.** 50 °C. **G.** 55 °C. **H.** 60 °C. **I.** 65 °C. **J.** 75 °C. **K.** 85 °C.

**Fig. 28:**
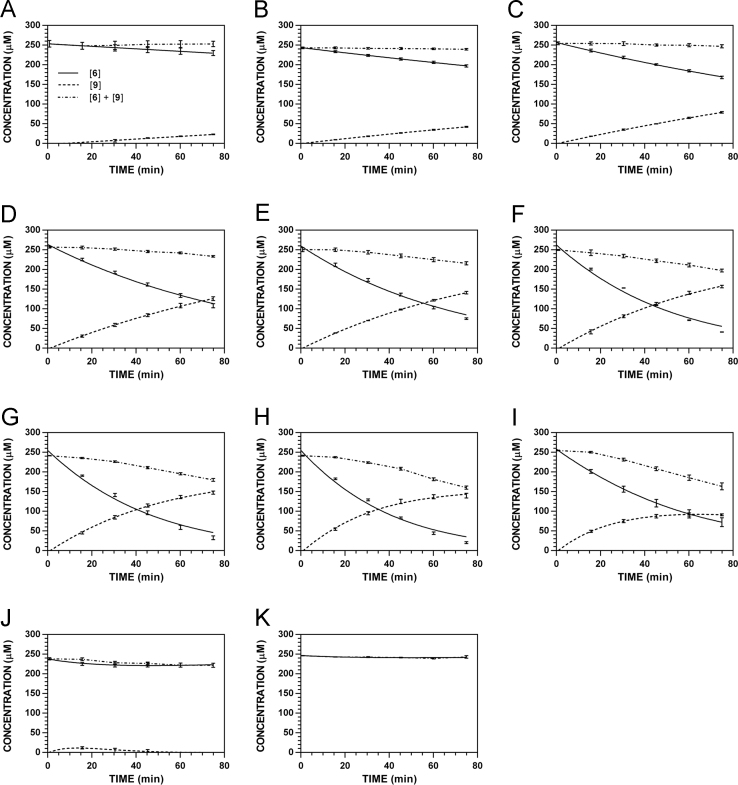
Reaction progress curves for the hydrolysis of **6** ([**6**]_0_ = 250 μM) at variable temperature and pH 7.4 (241 nm). **A.** 10.1 °C. **B.** 17.8 °C. **C.** 28.5 °C. **D.** 37 °C. **E.** 45 °C. **F.** 50 °C. **G.** 55 °C. **H.** 60 °C. **I.** 65 °C. **J.** 75 °C. **K.** 85 °C.

Fig. 22, Fig. 23, Fig. 24, Fig. 25, Fig. 26, Fig. 27, Fig. 28.

### 1.4 Tables of initial velocities

#### 1.4.1 Variable concentration of substrate

Table 1.

#### 1.4.2 Variable concentration of enzyme

Table 2.

#### 1.4.3 Variable pH

Table 3, Table 4, Table 5.

#### 1.4.4 Variable temperature

Table 6, Table 7.

## 2 Experimental design,materials and methods

### 2.1 General synthetic information

Synthetic reactions were performed using commercial reagents and materials under inert conditions, unless otherwise specified.

### 2.2 2Synthesis of 2,2-diphenylethyl isothiocyanate

**Scheme 1:**
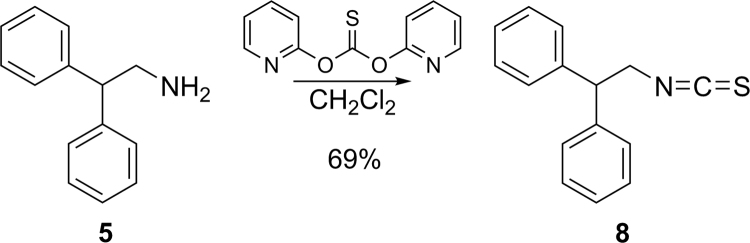
Preparation of 2,2-diphenylethyl ITC.

2,2-Diphenylethyl ITC (**8**) was prepared from reaction of its corresponding primary amine (**12**, Scheme 1) with di-2-pyridylthionocarbonate (D2PT) in moderate yield [2,^4^,5].

### 2.3 Synthesis of 2,2-diphenylethyl glucosinolate

**Scheme 2:**
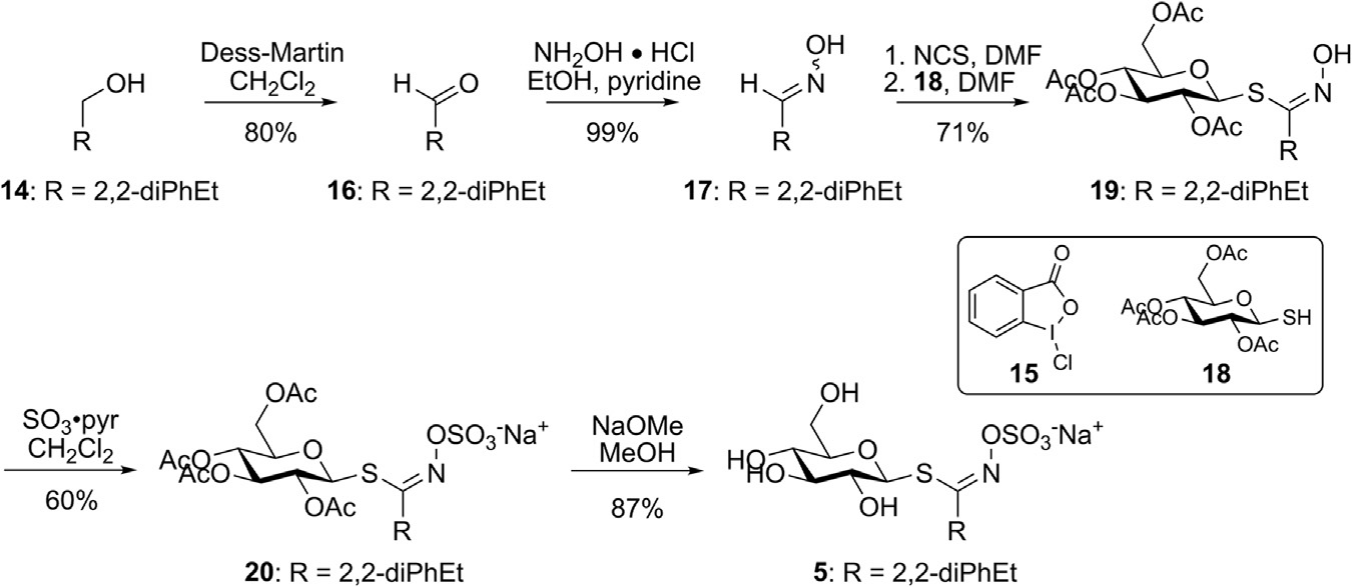
Preparation of 2,2-diphenylethyl glucosinolate.

Glucosinolate **5** was prepared from its corresponding alcohol (**14**) using the aldoxime method previously employed by our group (Scheme 2) [2,^4^,6]. Reagent **15** was prepared in high yield for minimal cost [7] and its use in the conversion of **14** to **16** was both high yielding and easy to purify. Condensation of aldehyde **16** with hydroxylamine afforded oxime **17** in high yield [6,8]. Treatment of **17** with *N*-chlorosuccinimide formed an intermediate oximyl chloride, which was immediately coupled to 2,3,4,6-tetra-*O*-acetyl-1-thio-β-D-glucose (**18**) to provide scaffold **19**. Sulfonation of **19** was accomplished with sulfur trioxide pyridine complex to afford intermediate **20**, which was deprotected via Zempelen *O*-deacetylation to provide glucosinolate **5** in high yield.

### 2.4 Synthetic experimentals

#### 2.4.1 Preparation of (2-isothiocyanatoethane-1,1-diyl)dibenzene (8)

To a solution of 2,2-diphenylethylamine (200 mg, 1.01 mmol) in dry CH_2_Cl_2_ (20.0 ml) at ambient temperature was added di(2-pyridyl)thionocarbonate (462 mg, 1.99 mmol). The reaction was stirred for 24 h and the solvent was concentrated. Flash chromatography (SiO_2_, 20:1 hexanes:EtOAc) afforded **8** as a colorless solid (168 mg, 69%): m.p. 36.2–36.5 °C; ^1^H NMR (CDCl_3_, 400 MHz) δ 7.38–7.32 (m, 4H), 7.31–7.21 (m, 5H), 4.38 (t, *J* = 7.7 Hz, 1H), 4.10 (d, *J* = 7.3 Hz, 2H); ^13^C NMR (CDCl_3_, 100 MHz) δ 140.4 (2C), 132.0, 129.1 (4C), 128.1 (4C), 127.6 (2C), 51.6, 49.6; IR (KBr) ν_max_ 3058, 3026, 2920, 2900, 2850, 2770, 2705, 2361, 2338, 2188, 2110, 1771, 1733, 1716, 1700, 1683, 1670, 1652, 1635, 1616, 1598, 1558, 1540, 1521, 1506, 1492, 1451, 1384, 1361, 1346, 1322, 1186, 1155, 1089, 1024, 968, 921 cm^−1^; HRMS (ESI+) *m/z*: [M + H]^+^ calcd for C_15_H_14_NS, 240.0847; found, 240.0842.

#### 2.4.2 Preparation of 3,3-diphenylpropanal (**16**)

To a solution of **14** (2.00 g, 9.42 mmol), TEMPO (0.12 g, 0.75 mmol), pyridine (2.26 ml, 28.26 mmol) in EtOAc (46 ml) was added **15** (3.99 g, 14.14 mmol) and was stirred at rt for 18 h. The oxidant was quenched with addition of saturated aqueous Na_2_S_2_O_3_ (10 ml) and was extracted with EtOAc (3×30 ml). The combined organic layers were washed with hydrochloric acid (1 M, 15 ml), water (15 ml), dried (Na_2_SO_4_), and concentrated. Flash chromatography (SiO_2_, 3:1 hexanes:CH_2_Cl_2_) afforded **16** as a colorless solid (1.65 g, 84%): m.p. 32.0–32.5 °C; ^1^H NMR (CDCl_3_, 400 MHz) δ 9.75 (t, *J* = 1.8 Hz, 1H), 7.34-7.28 (m, 4H), 7.27–7.19 (m, 5H), 4.64 (t, *J* = 7.8 Hz, 1H), 3.19 (dd, *J* = 7.8, 1.8 Hz, 2H); ^13^C NMR (CDCl_3_, 100 MHz) δ 201.3, 143.4 (2C), 128.9 (2C), 127.9 (2C), 126.9 (2C), 49.6, 45.2; IR (KBr) ν_max_ 3083, 3062, 3027, 2880, 2861, 2839, 2737, 1920, 1900, 1870, 1716, 1599, 1583, 1494, 1451, 1405, 1388, 1180, 1089, 1055, 1032, 926, 748 cm^−1^; HRMS (ESI+) *m/z*: [M]^+^ calcd for C_15_H_14_O, 210.1045; found, 210.1022.

#### 2.4.3 Preparation of 3,3-diphenylpropanal oxime (**17**)

Hydroxylamine hydrochloride (466 mg, 6.71 mmol) was added to **16** (659 mg, 3.13 mmol), EtOH (95%, 13.0 ml), and pyridine (1.30 ml, 16.10 mmol). The solution was heated to reflux for 3 h, then the solvents were concentrated. The residue was dissolved in water:EtOAc (1:1, 150 ml) and the aqueous layer was extracted with EtOAc (3×50 ml). The combined organic layers were washed with saturated aqueous sodium chloride (150 ml), dried (Na_2_SO_4_), and concentrated to afford **17** as a colorless solid in a 1:1 ratio of *E*:*Z* isomers (710 mg, 100%): m.p. 81.2–83.0 °C; ^1^H NMR (CDCl_3_, 400 MHz) δ 7.37-7.18 (m, 22H), 6.99 (s, 1H), 6.67 (t, *J* = 5.5 Hz, 1H), 4.24 (t, *J* = 8.2 Hz, 1H), 4.19 (t, *J* = 7.8 Hz, 1H), 3.14 (dd, *J* = 8.2, 5.0 Hz, 2H), 2.97 (dd, *J* = 7.8, 6.0 Hz, 2H); ^13^C NMR (CDCl_3_, 100 MHz) δ 151.5, 151.2, 143.8, 143.6, 128.9, 128.0, 126.8, 49.1, 48.2, 35.5, 31.0; IR (KBr) ν_max_ 3252, 3084, 3060, 3053, 3042, 3026, 2942, 2923, 2892, 2873, 2853, 1662, 1597, 1493, 1445, 1344, 1233, 1172, 1091, 1064, 1022, 918, 837, 719, 693, 631, 580, 537, 459 cm^−1^; HRMS (ESI+) *m/z*: [M + H]^+^ calcd for C_15_H_16_NO, 226.1232; found, 226.1247.

#### 2.4.4 Preparation of (2R,3R,4S,5R,6S)-2-(acetoxymethyl)-6-((Z)-1-(hydroxyimino)-3,3-diphenylpropylthio)tetrahydro-2H-pyran-3,4,5-triyl triacetate (**19**)

Compound **17** (650 mg, 2.89 mmol) was dissolved in dry DMF (19.0 ml) and NCS (384 mg, 2.88 mmol) was added slowly over 10 min. The solution was heated to 75 °C for 2 h. 2,3,4,6-Tetra-*O*-acetyl-1-thio-β-D-glucose (1.098 g, 3.01 mmol), and *N*,*N*-diisopropylethylamine (4.50 ml, 23.94 mmol) were added, and the reaction was stirred for 18 h. The reaction was diluted with EtOAc (75 ml) and washed with H_2_SO_4_ (1M, 150 ml). The aqueous phase was extracted with EtOAc (3×50 ml). The combined organic layers were washed with H_2_SO_4_ (1M, 100 ml), water (6×75 ml), dried (MgSO_4_), and concentrated. Flash chromatography (SiO_2,_ 6:3:1 hexanes:CH_2_Cl_2_:MeOH) afforded **19** as a colorless solid (1.21 g, 71%): m.p. 82.1–85.1 °C; ^1^H NMR (CDCl_3_, 400 MHz) δ 8.08 (s, 1H), 7.34–7.17 (m, 10H), 5.20 (t, *J* = 9.2 Hz, 1H), 5.08 (d, *J* = 10.1 Hz, 1H), 5.03 (d, *J* = 8.7 Hz, 1H), 4.94 (d, *J* = 10.1 Hz, 1H), 4.56 (t, *J* = 7.8 Hz, 1H), 4.16 (dd, *J* = 12.8, 6.0 Hz, 1H), 4.07 (dd, *J* = 12.4, 2.3 Hz, 1H), 3.65 (ddd, *J* = 10.1, 6.0, 2.3 Hz, 1H), 3.34 (dd, *J* = 15.6, 7.8 Hz, 1H), 3.19 (dd, *J* =15.1, 7.3 Hz, 1H), 2.07 (s, 3H), 2.03 (s, 3H), 2.02 (s, 3H), 1.95 (s, 3H); ^13^C NMR (CDCl_3_, 100 MHz) δ ; I170.9, 170.4, 169.6, 169.4, 150.1, 144.0, 143.3, 128.9 (2C), 128.8 (2C), 128.0 (2C), 127.9 (2C), 126.9, 126.8, 80.3, 76.2, 73.8, 70.2, 68.1, 62.4, 48.4, 38.9, 20.9, 20.8, 20.8 (2C); IR (KBr) ν_max_ 3420, 3163, 3028, 2360, 2341, 1751, 1452, 1399, 1384, 1225, 1039, 702, 668 cm^−1^; HRMS (ESI+) *m/z*: [M + Na]^+^ calcd for C_29_H_33_NO_10_SNa, 610.1723; found, 610.1727.

#### 2.4.5 Preparation of sodium (Z)-3,3-diphenyl-1-((2S,3R,4S,5R,6R)-3,4,5-triacetoxy-6-(acetoxymethyl)tetrahydro-2H-pyran-2-ylthio)propylideneamino sulfate (**20**)

To a solution of sulfur trioxide/pyridine complex (4.85 g, 30.50 mmol) in dry CH_2_Cl_2_ (200 ml) was added **19** (3.58 g, 6.10 mmol) in CH_2_Cl_2_ (200 ml). After 48 h, saturated aqueous sodium bicarbonate (90 ml) was slowly added, and the solvents were concentrated. Flash chromatography (SiO_2_, 6:3:1 pet ether:CH_2_Cl_2_:MeOH), afforded **20** as a pale yellow solid (2.24 g, 60%): m.p. 139.0 °C (decomposed); ^1^H NMR (CD_3_OD, 400 MHz) δ 7.38 (m, 4H), 7.30 (m, 4H), 7.20 (m, 2H), 5.27 (t, *J* = 9.6 Hz, 1H), 5.10 (d, *J* = 10.1 Hz, 1H), 5.02 (t, *J* = 9.6 Hz, 1H), 4.94 (t, *J* = 9.6 Hz, 1H), 4.73 (t, *J* = 8.2 Hz, 1H), 4.19 (dd, *J* = 12.4, 6.0 Hz, 1H), 4.12 (dd, *J* = 12.8, 2.3 Hz, 1H), 3.94 (ddd, *J* = 10.1, 6.0, 2.3 Hz, 1H), 3.46 (dd, *J* = 15.6, 6.4 Hz, 1H), 3.39 (dd, *J* = 15.6, 8.2 Hz, 1H), 2.04 (s, 3H), 2.03 (s, 3H), 1.99 (s, 3H), 1.90 (s, 3H); ^13^C NMR (CD_3_OD, 100 MHz) δ 172.3, 171.4, 171.2, 170.9, 157.2, 145.4, 145.1, 129.6, (2C), 129.5 (4C), 129.1 (2C), 127.7, 127.5, 80.9, 76.9, 74.9, 71.1, 69.6, 63.7, 49.5, 39.7, 20.6 (2C), 20.5, 20.5; IR (film) ν_max_ 3502, 3063, 3029, 2941, 1756, 1601, 1584, 1497, 1453, 1435, 1383, 1230, 1062, 1041, 911, 898, 798 cm^−1^.

#### 2.4.6 Preparation of sodium (Z)-3,3-diphenyl-1-((2S,3R,4S,5S,6R)-3,4,5-trihydroxy-6-(hydroxymethyl)tetrahydro-2H-pyran-2-ylthio)propylideneamino sulfate (5)

To a solution of **20** (1.91 g, 2.77 mmol) in dry MeOH (38 ml) was added NaOMe in MeOH (1 M, 1.91 ml). The solution was stirred at rt for 2 h, then acetic acid (207 μL) was added. The solvents were concentrated and the residue purified by flash chromatography (SiO_2_, 4:1 EtOAc:MeOH) to afford **5** as a colorless solid (1.25 g, 87%): m.p. 180 °C (decomposed); ^1^H NMR (CD_3_OD, 400 MHz) δ 7.32 (m, 4H), 7.23 (td, *J* = 7.3, 3.2 Hz, 4H), 7.12 (m, 2H), 4.71–4.65 (m, 2H), 3.81 (dd, *J* = 11.0, 1.8 Hz, 1H), 3.62 (dd, *J* = 12.4, 6.0 Hz, 1H), 3.49 (dd, *J* = 15.1, 7.6 Hz, 1H), 3.39 (dd, *J* = 15.6, 7.3 Hz, 1H), 7.34–7.18 (m, 4H); ^13^C NMR (CD_3_OD, 100 MHz) δ 159.9, 145.6, 144.9, 129.5 (2C), 129.3 (2C), 129.1 (4C), 127.4 (2C), 127.2 (2C), 83.7, 82.1, 79.3, 74.0, 70.9, 62.4, 49.3, 39.8; IR (KBr) ν_max_ 3416, 2923, 2543, 1717, 1600, 1496, 1453, 1384, 1241, 1061, 956, 889, 804 cm^−1^.

### 2.5 Standardization

The specific activity of commercial *Sinapis alba* myrosinase was determined using the prescribed method [9]. Each analyte was individually standardized using the previously-described HPLC method [2], with minor modifications appropriate to the current related study [1]. Standard curves representing peak area vs. injection amount were generated for each wavelength of interest.

### 2.6 Generation of reaction progress curves and reaction velocities

Enzymatic hydrolysis reactions of glucosinolates were conducted in aqueous buffer using a modified form of the established protocol [1,2]. The concentration of glucosinolate, concentration of *Sinapis alba* myrosinase, buffer pH, and incubation temperature were varied for a given experiment, which were conducted in triplicate. Analytes at a given reaction timepoint were separated by HPLC and concentrations were determined from integration of analyte peak areas. Reaction progress curves were fit to the data using a modified form of the Lambert *W*(x) and were used to calculate initial rates of hydrolysis/formation for each observed analyte [1].

## Figures and Tables

**Fig. 1:**
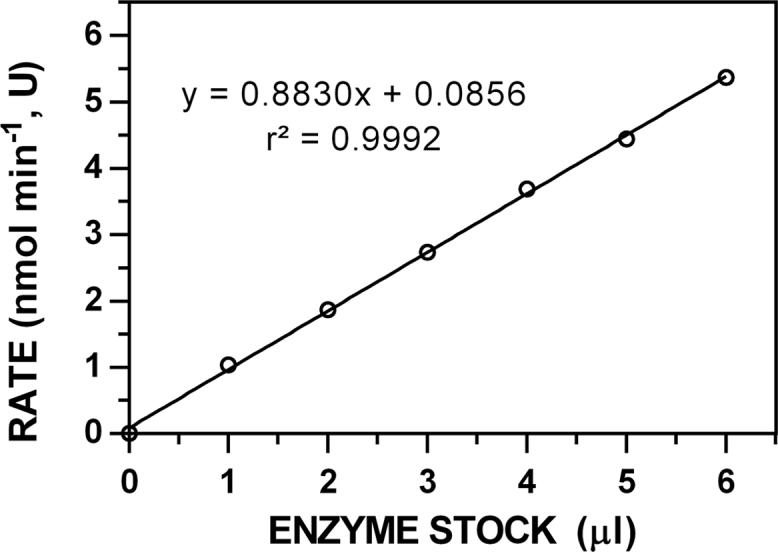
Representative data to determine the specific activity of 10 mg ml^−1^ myrosinase stock solutions. Rates were determined for the hydrolysis of sinigrin ([**7**]_0_ = 250 μM) at 227 nm (Δε_227_ = 6458 M^−1^ cm^−1^) for 5 min [2,3].

**Fig. 2:**
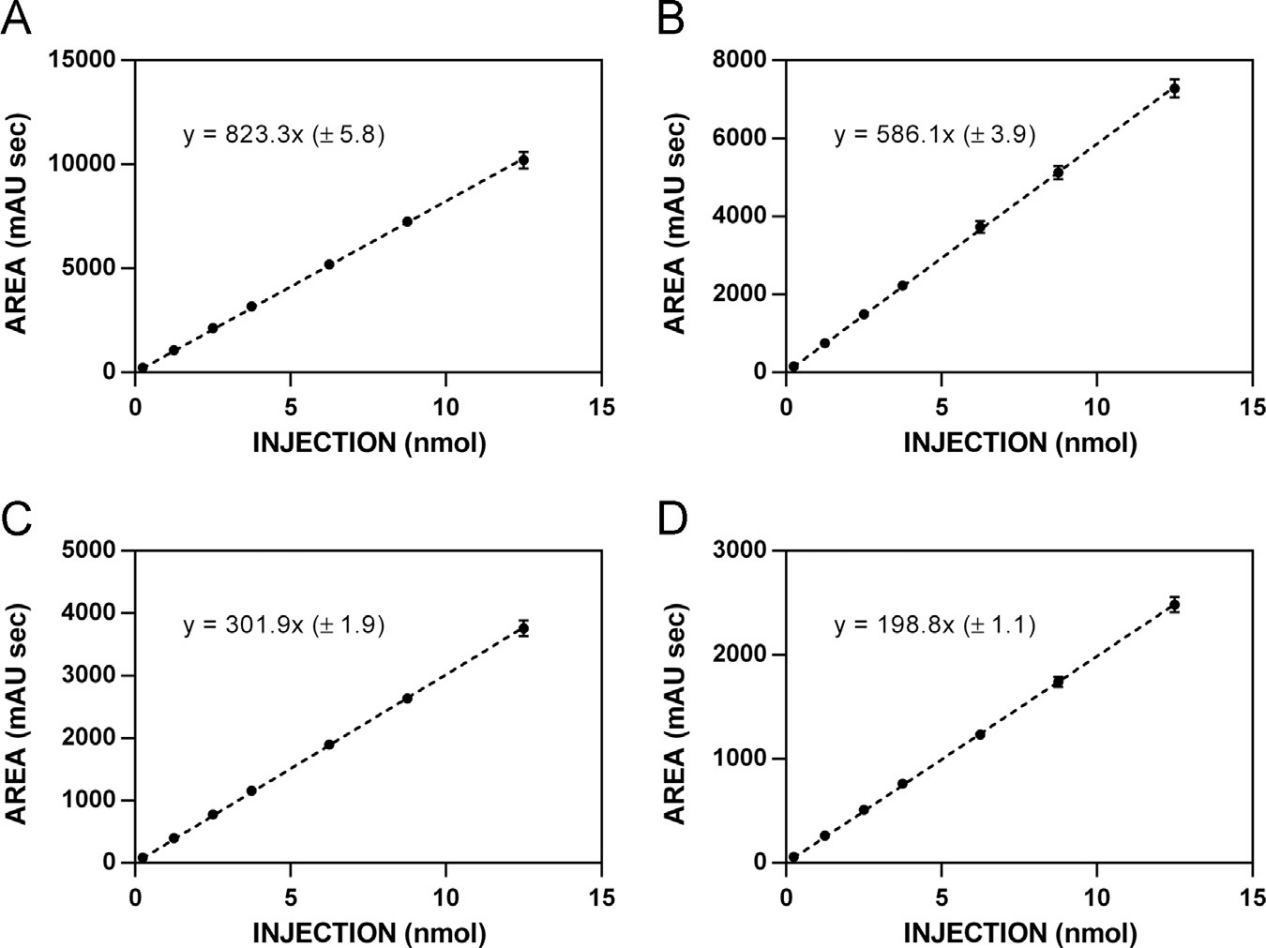
HPLC standardization curves for glucosinolate **5. A**. 220 nm. **B**. 227 nm. **C**. 235 nm. **D**. 241 nm.

**Fig. 3:**
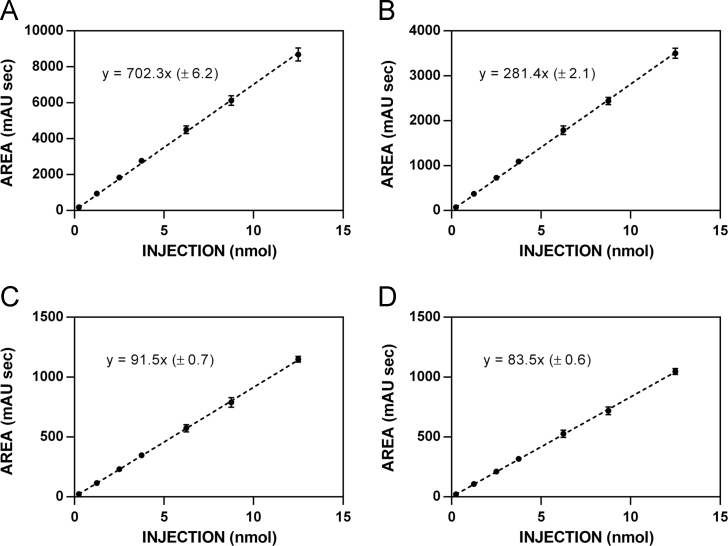
HPLC standardization curves for isothiocyanate **8. A**. 220 nm. **B**. 227 nm. **C**. 235 nm. **D**. 241 nm.

**Fig. 4:**
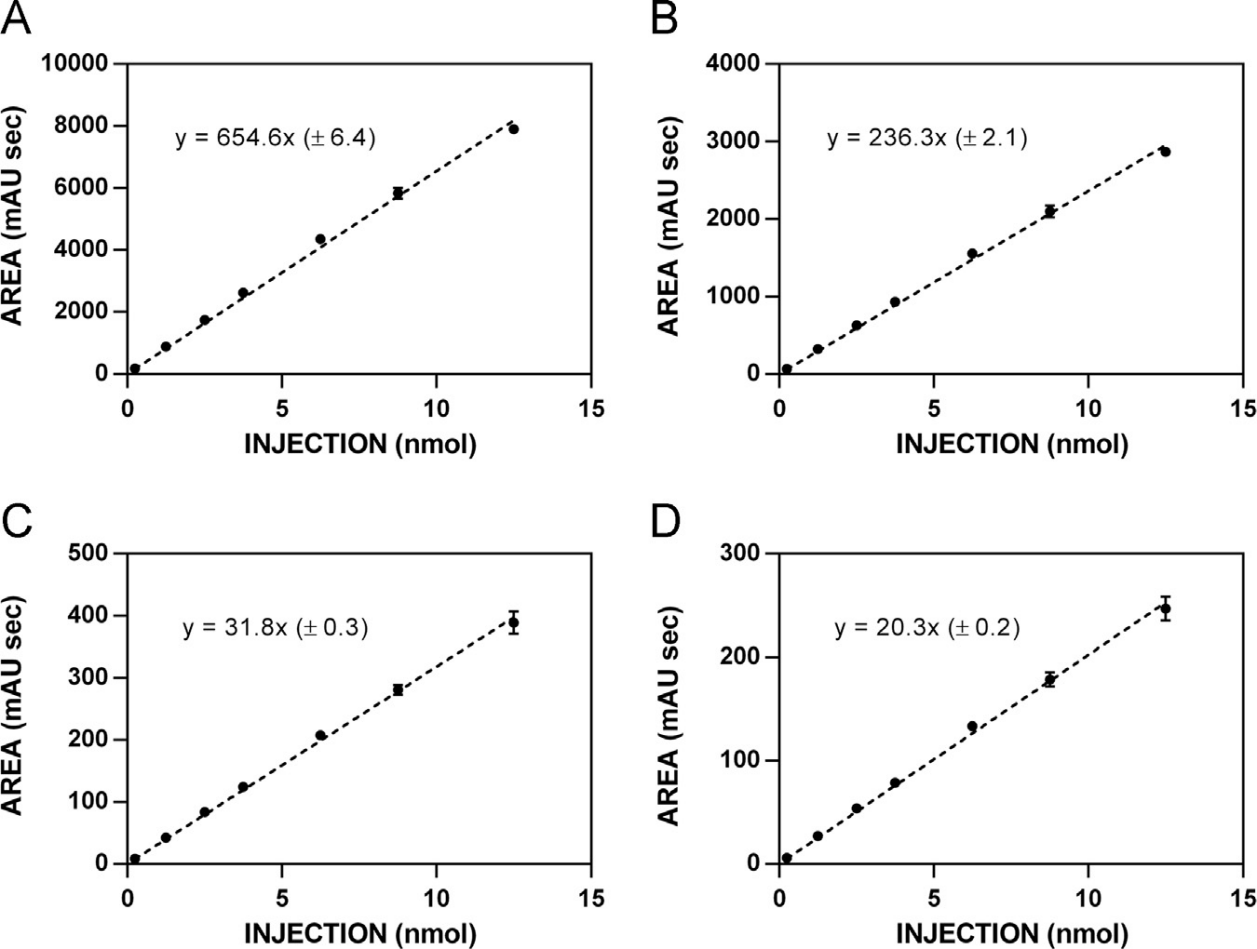
HPLC standardization curves for nitrile **10. A**. 220 nm. **B**. 227 nm. **C**. 235 nm. **D**. 241 nm.

**Fig. 5:**
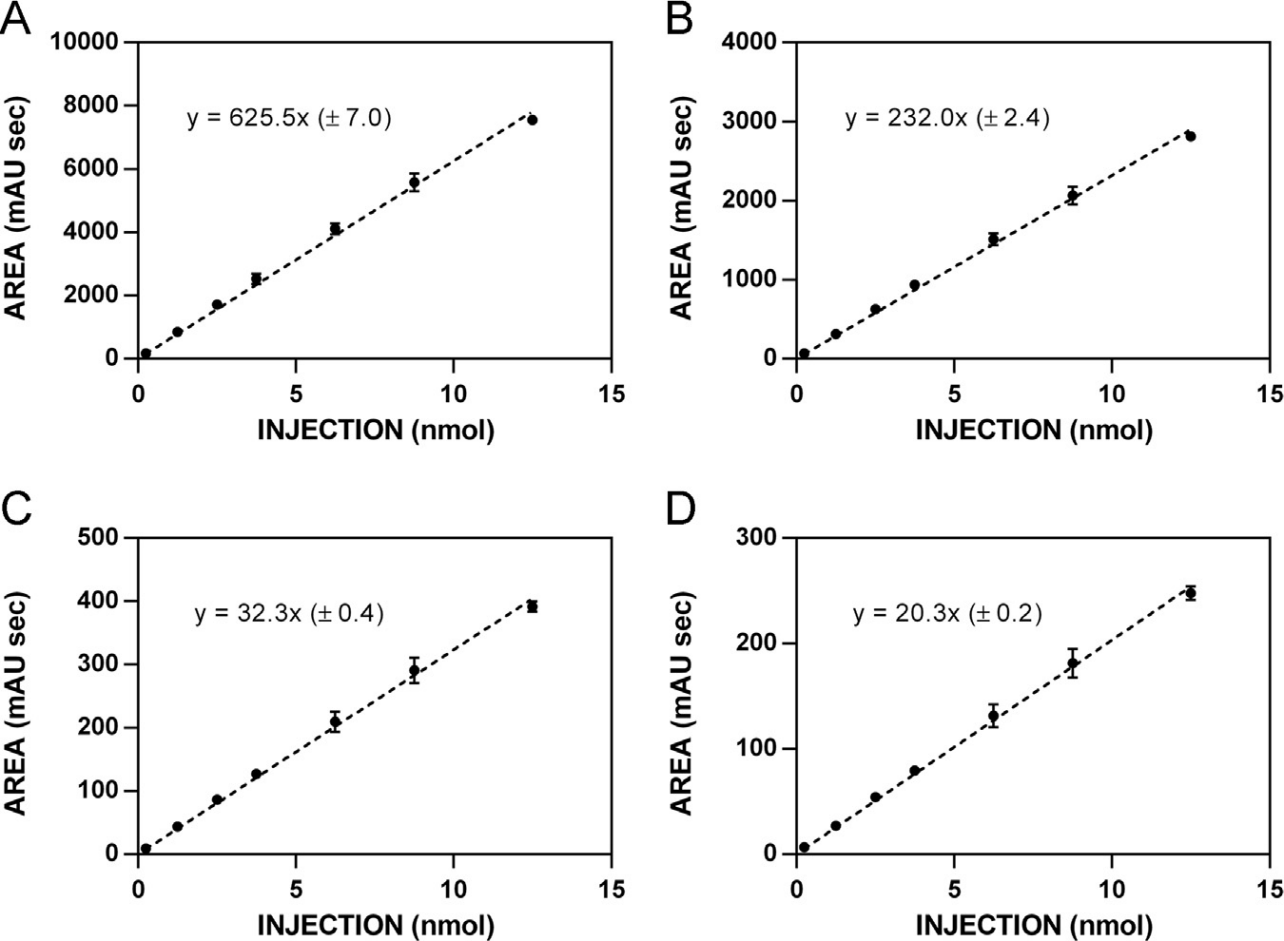
HPLC standardization curves for amine **12. A**. 220 nm. **B**. 227 nm. **C**. 235 nm. **D**. 241 nm.

**Fig. 6:**
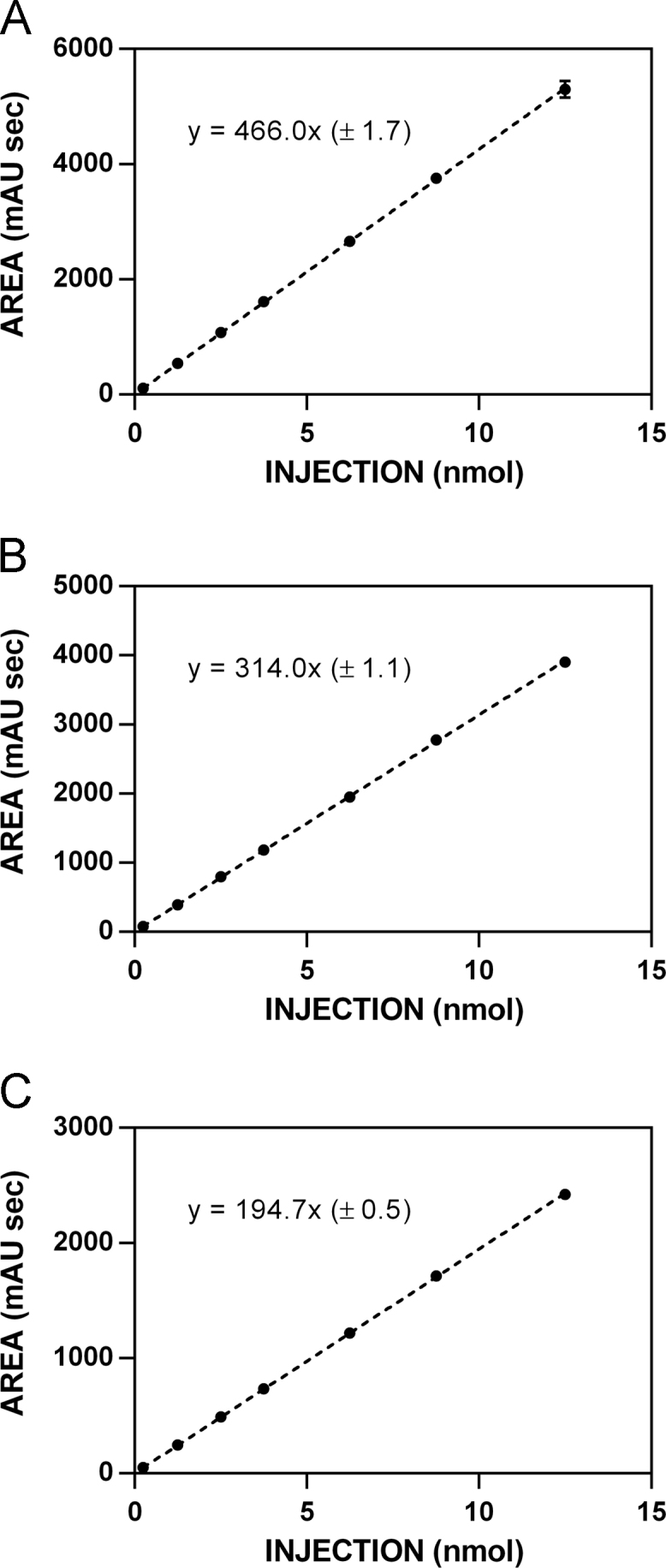
HPLC standardization curves for glucosinolate **6. A**. 227 nm. **B**. 235 nm. **C**. 241 nm.

**Fig. 7:**
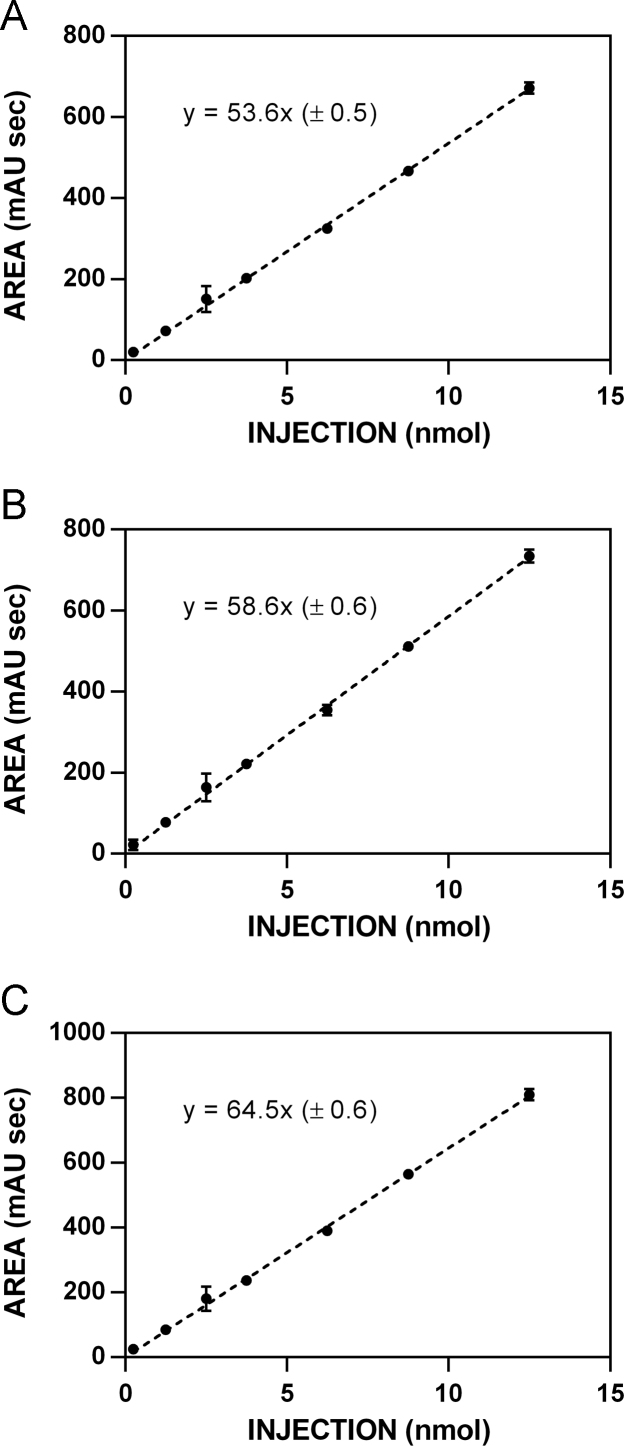
HPLC standardization curves for isothiocyanate **9. A**. 227 nm. **B**. 235 nm. **C**. 241 nm.

**Fig. 8:**
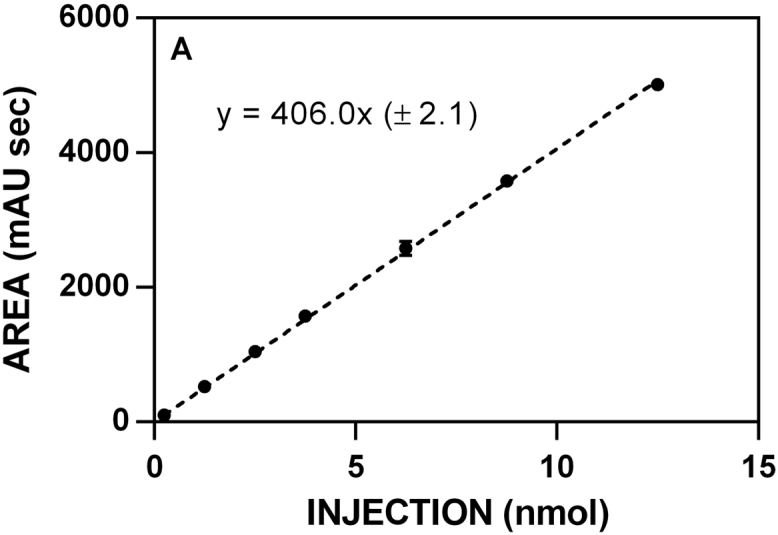
HPLC standardization curve for nitrile **11. A**. 210 nm.

**Fig. 9:**
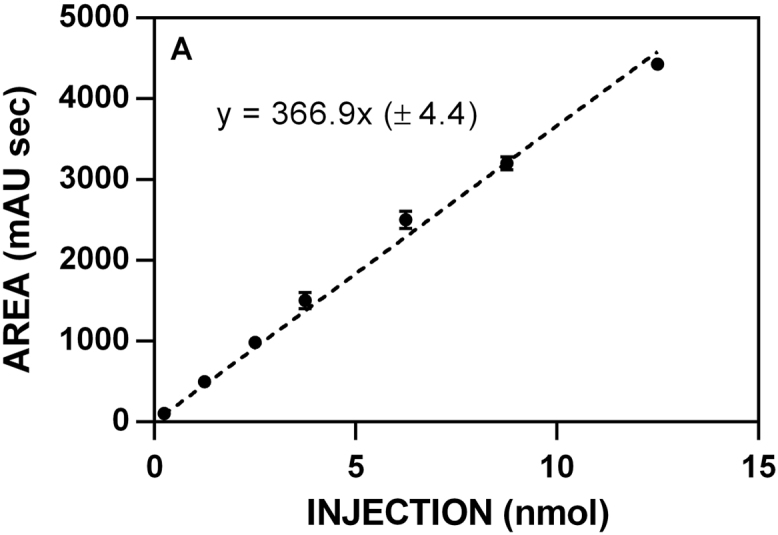
HPLC standardization curve for amine **13. A**. 210 nm.

**Fig. 14:**
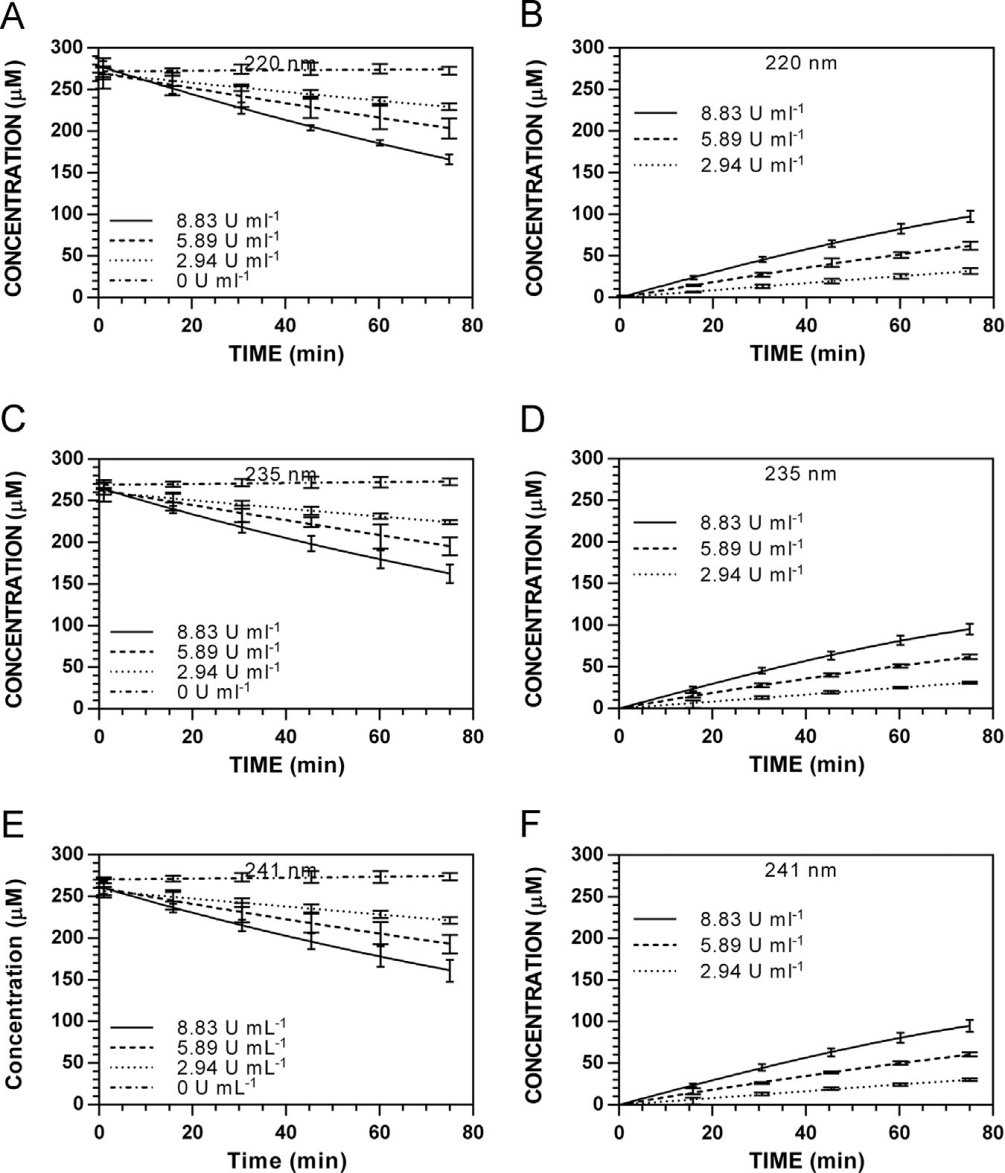
Enzyme-dependence on reaction progress curves for [**5**]_t_ and [**8**]_t_ at pH 7.4 and 37 °C ([**5**]_0_ = 250 μM). **A.** [**5**]_t_, 220 nm. **B.** [**8**]_t_, 220 nm. **C.** [**5**]_t_, 235 nm. **D.** [**8**]_t_, 235 nm. **E.** [**5**]_t_, 241 nm. **F.** [**5**]_t_, 241 nm.

**Table 1:** Initial rates of hydrolysis of **5** and observed formation of **8** (pH 7.4, 37 °C) by *Sinapis alba* myrosinase. The concentration of myrosinase was constant (8.83 U ml^−1^).

**[Gluc]**_**0**_**(μM)**	**Δ[5] Δt**^**−1**^**(μM min**^**−1**^**)**			
	**220 nm**	**227 nm**	**235 nm**	**241 nm**
**1000**	−4.11	−4.24	−4.16	−4.31
**500**	−2.74	−2.82	−2.89	−2.77
**250**	−1.75	−1.70	−1.67	−1.64
**125**	−1.12	−1.06	−1.07	−1.03
**62.5**	−0.65	−0.66	−0.71	−0.70
**31.3**	−0.46	−0.48	−0.51	−0.48
	**Δ[8]**_**obs**_**Δt**^−1^**(μM min**^−1^**)**			
**1000**	4.48	4.83	4.65	4.76
**500**	2.70	2.99	3.89	3.54
**250**	1.63	1.67	1.71	1.68
**125**	0.96	1.00	1.10	1.05
**62.5**	0.62	0.72	0.74	0.67
**31.3**	0.45	0.46	0.49	0.47

**Table 2:** Enzyme-dependence on the rate of conversion for **5** ([**5**]_0_ = 250 μM) to **8** (pH 7.4, 37 °C). The concentration of myrosinase ([Myr]) was 100%, 67%, 33%, and 0% of a maximum value (8.83 U ml^−1^). Reaction progress curves for [ITC]_t_ with 0% [Myr] were not generated due to negligible levels of detected [ITC] [1].

**[Myr] (relative)**	**Δ[5] Δt**^**−1**^**(μM min**^**−1**^**)**			
	**220 nm**	**227 nm**	**235 nm**	**241 nm**
**100%**	−1.75	−1.70	−1.67	−1.64
**67%**	−0.95	−0.93	−0.91	−1.01
**33%**	−0.58	−0.57	−0.53	−0.48
**0%**	0.03	0.06	0.05	0.00
	**Δ[8]**_**obs**_**Δt**^−1^**(μM min**^−1^**)**			
**100%**	1.63	1.67	1.71	1.68
**67%**	0.96	0.89	1.00	0.96
**33%**	0.47	0.46	0.45	0.46

**Table 3:** pH Dependence of the action of *Sinapis alba* myrosinase on the rate constant for glucosinolate hydrolysis ([Gluc]_0_ = 250 μM, 37 °C).

**pH**	**Δ[5] Δt**^**−1**^**[Myr]**^**−1**^**(min**^**−1**^**)**				**Δ[6] Δt**^**−1**^**[Myr]**^**−1**^**(min**^**−1**^**)**		
	**220 nm**	**227 nm**	**235 nm**	**241 nm**	**227 nm**	**235 nm**	**241 nm**
**2.0**	0.00	0.00	0.00	0.00	0.01	0.00	0.00
**3.0**	0.27	0.26	0.27	0.28	0.95	0.95	0.97
**4.0**	0.43	0.43	0.43	0.43	1.27	1.23	1.28
**5.0**	0.40	0.38	0.36	0.36	1.18	1.27	1.30
**6.0**	0.34	0.34	0.34	0.33	1.28	1.23	1.19
**7.4**	0.20	0.19	0.19	0.19	1.23	1.25	1.27
**8.0**	0.22	0.18	0.19	0.22	1.21	1.19	1.22
**9.0**	0.17	0.17	0.17	0.18	1.10	1.07	1.15
**10.0**	0.13	0.12	0.14	0.12	1.07	1.05	1.08

**Table 4:** pH Dependence of the action of *Sinapis alba* myrosinase on the rate constant for observed ITC formation ([Gluc]_0_ = 250 μM, 37 °C).

**pH**	**Δ[8]**_**obs**_**Δt**^**−1**^**[Myr]**^**−1**^**(min**^**−1**^**)**				**Δ[9]**_**obs**_**Δt**^**−1**^**[Myr]**^**−1**^**(min**^**−1**^**)**		
	**220 nm**	**227 nm**	**235 nm**	**241 nm**	**227 nm**	**235 nm**	**241 nm**
**2.0**	0.00	0.00	0.00	0.00	0.00	0.00	0.00
**3.0**	0.08	0.09	0.09	0.08	0.40	0.41	0.39
**4.0**	0.32	0.31	0.33	0.33	1.07	1.09	1.09
**5.0**	0.34	0.34	0.35	0.35	1.29	1.31	1.31
**6.0**	0.31	0.31	0.33	0.32	1.15	1.16	1.16
**7.4**	0.18	0.19	0.19	0.19	1.23	1.28	1.27
**8.0**	0.20	0.18	0.19	0.18	1.31	1.37	1.27
**9.0**	0.17	0.16	0.17	0.17	1.08	1.11	1.10
**10.0**	0.16	0.15	0.18	0.16	0.98	1.03	1.03

**Table 5:** pH Dependence of the action of *Sinapis alba* myrosinase on the rate constant for observed nitrile formation ([Gluc]_0_ = 250 μM, 37 °C).

**pH**	**Δ[10]**_**obs**_**Δt**^**−1**^**[Myr]**^**−1**^**(min**^**−1**^**)**				**Δ[11]**_**obs**_**Δt**^**−1**^**[Myr]**^**−1**^**(min**^**−1**^**)**
	**220 nm**	**227 nm**	**235 nm**	**241 nm**	**210 nm**
**2.0**	0.00	0.00	0.00	0.00	0.00
**3.0**	0.18	0.18	0.16	0.14	0.54
**4.0**	0.11	0.11	0.11	0.08	0.18
**5.0**	0.02	0.03	0.01	0.00	0.00
**6.0**	0.00	0.00	0.00	0.00	0.00
**7.4**	0.00	0.00	0.00	0.00	0.00
**8.0**	0.00	0.00	0.00	0.00	0.00
**9.0**	0.00	0.00	0.00	0.00	0.00
**10.0**	0.00	0.00	0.00	0.00	0.00

**Table 6:** Temperature dependence of the action of *Sinapis alba* myrosinase on the rate constant for glucosinolate hydrolysis ([Gluc]_0_ = 250 μM, 37 °C).

	**Δ[5] Δt**^**−1**^**[Myr]**^**−1**^**(min**^**−1**^**)**				**Δ[6] Δt**^**−1**^**[Myr]**^**−1**^**(min**^**−1**^**)**		
**Temperature**	**220 nm**	**227 nm**	**235 nm**	**241 nm**	**227 nm**	**235 nm**	**241 nm**
**5**	0.01	0.01	0.01	0.01	0.20	0.19	0.21
**15**	0.03	0.02	0.03	0.02	0.39	0.40	0.39
**25**	0.08	0.08	0.06	0.06	0.79	0.79	0.79
**35**	0.17	0.17	0.17	0.17	1.59	1.58	1.61
**45**	0.30	0.31	0.29	0.29	2.11	2.06	2.07
**50**					2.80	2.79	2.82
**55**	0.61	0.60	0.60	0.58	2.92	2.87	2.98
**60**	0.74	0.73	0.70	0.71	3.39	3.31	3.39
**65**	0.89	0.82	0.87	0.85	2.28	2.24	2.24
**70**	0.29	0.25	0.27	0.28			
**75**	0.07	0.07	0.08	0.07	0.55	0.54	0.57
**85**					0.02	0.04	0.04

**Table 7:** Temperature dependence of the action of *Sinapis alba* myrosinase on the rate constant for observed ITC formation ([Gluc]_0_ = 250 μM, 37 °C).

**Temperature**	**Δ[8]**_**obs**_**Δt**^**−1**^**[Myr]**^**−1**^**(min**^**−1**^**)**				**Δ[9]**_**obs**_**Δt**^**−1**^**[Myr]**^**−1**^**(min**^**−1**^**)**		
	**220 nm**	**227 nm**	**235 nm**	**241 nm**	**227 nm**	**235 nm**	**241 nm**
**5**	0.02	0.01			0.20	0.20	0.20
**15**	0.02	0.02	0.02	0.02	0.35	0.36	0.37
**25**	0.05	0.06	0.06	0.06	0.69	0.70	0.70
**35**	0.16	0.16	0.16	0.16	1.22	1.30	1.28
**45**	0.28	0.28	0.28	0.29	1.55	1.58	1.58
**50**					1.61	1.84	1.89
**55**	0.59	0.60	0.59	0.59	2.04	2.08	2.10
**60**	0.74	0.74	0.75	0.74	2.73	2.78	2.75
**65**	0.84	0.84	0.79	0.79			
